# Regime Shift by an Exotic Nitrogen-Fixing Shrub Mediates Plant Facilitation in Primary Succession

**DOI:** 10.1371/journal.pone.0123128

**Published:** 2015-04-02

**Authors:** Adriano Stinca, Giovanni Battista Chirico, Guido Incerti, Giuliano Bonanomi

**Affiliations:** Department of Agriculture, University of Naples Federico II, via Università 100, Portici (Naples), Italy; National University of Mongolia, MONGOLIA

## Abstract

Ecosystem invasion by non-native, nitrogen-fixing species is a global phenomenon with serious ecological consequences. However, in the Mediterranean basin few studies addressed the impact of invasion by nitrogen-fixing shrubs on soil quality and hydrological properties at local scale, and the possible effects on succession dynamics and ecosystem invasibility by further species. In this multidisciplinary study we investigated the impact of *Genista aetnensis* (Biv.) DC., an exotic nitrogen-fixing shrub, on the Vesuvius Grand Cone (Southern Italy). Specifically, we tested the hypotheses that the invasion of *G*. *aetnensis* has a significant impact on soil quality, soil hydrological regime, local microclimate and plant community structure, and that its impact increases during the plant ontogenetic cycle. We showed that *G*. *aetnensis*, in a relatively short time-span (i.e. ~ 40 years), has been able to build-up an island of fertility under its canopy, by accumulating considerable stocks of C, N, and P in the soil, and by also improving the soil hydrological properties. Moreover, *G*. *aetnensis* mitigates the daily range of soil temperature, reducing the exposure of coexisting plants to extremely high temperatures and water loss by soil evaporation, particularly during the growing season. Such amelioration of soil quality, coupled with the mitigation of below-canopy microclimatic conditions, has enhanced plant colonization of the barren Grand Cone slopes, by both herbaceous and woody species. These results suggest that the invasion of *G*. *aetnensis* could eventually drive to the spread of other, more resource-demanding exotic species, promoting alternative successional trajectories that may dramatically affect the local landscape. Our study is the first record of the invasion of *G*. *aetnensis*, an additional example of the regime shifts driven by N-fixing shrubs in Mediterranean region. Further studies are needed to identity specific management practices that can limit the spread and impacts of this species.

## Introduction

Ecosystem invasion by non-native species is a global phenomenon with serious consequences for ecological, economic, and social systems [1]. Plant biological invasions are considered to be among the most serious threats to local biodiversity and ecosystem functioning in terrestrial ecosystems [2]. In this context, it is noteworthy that nitrogen (N)-fixing species are disproportionately represented in the lists of the most noxious invasive plants [3]. Remarkable examples include the spread of *Myrica faya* Dryand. on the Hawaii islands [4], *Mimosa pigra* L. in Australia [5], *Lupinus arboreus* Sims in Californian sand dunes [6], *Cytisus scoparius* (L.) Link in western USA grasslands [7], *Acacia longifolia* (Andrews) Willd. in European sand dunes [8], and *Spartium junceum* L. in America (e.g. [9,10]) and South Africa [11]. Invasive plant species can affect ecosystem functions by increasing or decreasing fire frequency and intensity [12], changing hydrologic and geomorphic processes [13], and altering nutrients cycling [14]. Nitrogen-fixing invasive species, by directly accessing atmospheric nitrogen, can increase N availability in invaded ecosystems [15] and therefore are expected to be especially effective in changing carbon (C) and N cycling. In fact, these trees and shrubs, by fixing atmospheric N and tracking nutrients from the surrounding soil, progressively lead to a local nutrient recycling with N and organic C accumulation [16]. The resulting resource distribution, also called “islands of fertility” [17], often drives to facilitative interactions for the establishment and reproduction of coexisting species (review in [18,19]).

Observations of plant induced “islands of fertility” are now widespread and have been reported for several N-fixing species such as *Olneya tesota* A. Gray [20], *Acacia* spp. (e.g. [21]), *Mimosa luisana* Brandegee [22], *Medicago marina* L. [23], *Prosopis* spp. (e.g. [24]), *Retama sphaerocarpa* (L.) Boiss. [25] and *Ulex parviflorus* Pourr. [26] among many others. In spite of such evidence, data concerning the temporal dynamics of “fertility island” development under N-fixing plants are limited. A notable exception was the study by Facelli and Brock [27] that reported an increase of the amount of organic C, N, phosphorus (P) and sulphur (S) under the canopy of 150 years old *Acacia papyrocarpa* Benth. trees in the arid climate of South Australia. However, such kind of data are not available for Mediterranean climate areas. This is of particular concern because a higher soil fertility under the canopies of perennial nitrogen-fixing plants than in their neighbourhood, may favour the invasion of other, more resource-demanding invasive species [28]. However, both facilitative and competitive effects on coexisting plants have been reported for invasive N-fixing species [18]. Several studies reported a decrease of plant cover and species diversity under invasive canopy compared to adjacent non-invaded area, with changes in microclimate due to deep canopy shading proposed as putative causal mechanism [19]. The canopy shading reduces the incident solar radiation, contributing to mitigate soil and air temperature extremes, and consequently soil evaporation loss, as compared with open areas without vegetation (e.g. [29,30]). Soil water retention is also enhanced underneath the canopy, due to higher organic matter content, thus contributing to sustain higher soil water content and litter decomposition rate than in open areas [31,32]. Indeed, the interplay between facilitation and competition can shift during the ontogenetic life cycle of potential nurse plants [33,34]. In this regard, the competitive effects of invasive plants can turn into facilitative interactions after shrub death as a result of competition release both above- and below-ground [35]. Hence, a better understanding of how invasive species affect soil quality and local microclimate is of crucial importance to predict their effects on the structure of invaded plant communities [15].

**Fig 1:**
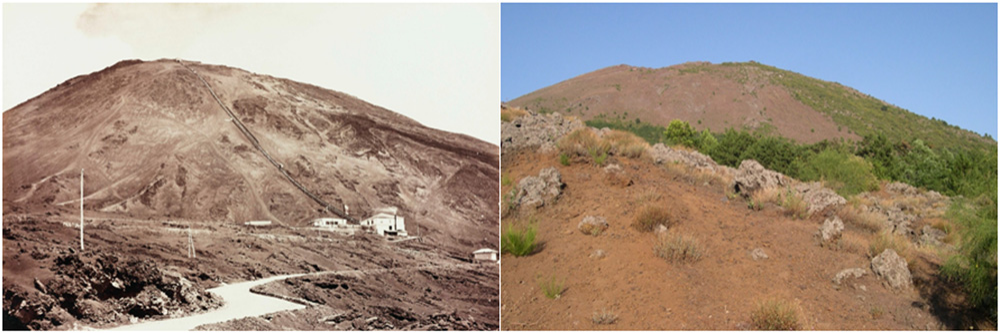
*Genista aetnensis* invasion of the Vesuvius Grand Cone. Changing landscape at the Vesuvius Grand Cone following *Genista aetnensis* invasion. Pictures have been taken from the West side of the Grand Cone in 1880 (Raccolte Museali Fratelli Alinari, Firenze, Italy) and in 2013 (Picture by Stinca A.).

Here, we explored whether and how plant-plant interactions, as well as plant capability to modify below- and above-ground environment, change during the ontogenetic development of an invasive shrubs. We tested this hypothesis by focusing on the *Genista aetnensis* (Biv.) DC., a nitrogen-fixing shrub that is rapidly invading the Mt. Vesuvius slopes (Fig 1), after being deliberately imported after the eruption of 1906 within a reforestation program, to mitigate soil erosion and increase slope stability [36].

Our hypothesis is that the invasion of *G*. *aetnensis* has a significant ecosystem impact, likely increasing during the plant ontogenetic cycle, because it occurs on N poor, young volcanic sites [15]. Here, such hypothesis is addressed with a multidisciplinary approach by comparing soil quality, soil hydrological regime, local microclimate and plant community structure between two environmental settings differently affected by *G*. *aetnensis*: the area underneath the canopy crowns, and the outer area not previously colonized by invasive species, far apart from the closest individual. Since soil quality cannot be summarized by a single property or process, we used 19 complementary indicators, including physical, chemical, biochemical, biological and hydrological variables. Then we specifically tested the hypotheses that *G*. *aetnensis*, during its ontogenetic cycle:

1. rapidly modifies the belowground environment in the area under the canopy, locally promoting the development of a “fertility island”;
2. improves below-canopy microclimate by mitigating maximum air and soil temperatures;
3. facilitates the interactions with coexisting species by providing soil nutrients and ameliorating soil hydrological conditions, thus fostering primary succession.

## Materials and Methods

### Study area and target species

The experimental site is located on the east-facing side (slope ranging from 40° to 45°) of the Vesuvius Grand Cone (Southern Italy, 40°49’17” N -14°25’34” E, 850–1150 m a.s.l.). The substrate is a sandy, gravel rich andosols derived from unconsolidated pyroclastic deposits. The main plant community is derived from a primary succession process [37,38,39,40] over a bare and physically unstable substrate, composed by a fruticose lichen (*Stereocaulon vesuvianum* Pers.) and small perennials (*Rumex scutatus* L. subsp. *scutatus*, *Artemisia campestris* L. subsp. *variabilis* [Ten.] Greuter, *Scrophularia canina* L. subsp. *bicolor* [Sm.] Greuter, and *Centranthus ruber* [L.] DC. subsp. *ruber*). The area has a Mediterranean climate with a cold humid winter and a relatively hot, dry summer. Based on 20 years of meteorological data retrieved from a station located at the Mt. Vesuvius Observatory (612 m a.s.l., about 2 km from the study site), the mean monthly temperatures range between 21.6°C (July) and 5.7°C (January), while the mean annual rainfall is 1100 mm, mostly occurring in autumn (399 mm) and winter (408 mm). The Somma-Vesuvius complex presents a good floristic diversity [41,42,43,44].

*Genista aetnensis*, hereafter referred to only as *Genista*, is a multi-stemmed nitrogen-fixing shrub native to the Mt. Etna in Sicily and some locality in Sardinia [45]. It was imported in the survey area one century ago within a reforestation program to mitigate soil erosion and increase slope stability [36]. Nowadays, *Genista* has become the dominant plant species on the Vesuvius Grand Cone (Fig 1), reaching a cover of about 20%, forming continuous plant communities of shrubs with loose canopies of diameter up to 3 m and crown height up to 5 m [40].

### Field measurements

Field measurements were carried out to: (1) quantify the effects of *Genista* on soil quality in relation to its ontogenetic cycle; (2) assess the effect of *Genista* canopy on above- and below-ground microclimate and soil hydrology; (3) evaluate the effects of *Genista* on both the distribution of coexisting species and the structure of local plant community. The Vesuvius National Park authority allowed the field surveying and sampling in the study site.

#### *Genista* ontogenetic stages and sampling strategy

During our field campaigns, we used a stratified random sampling design, distributed in two areas subjected respectively to a different degree of potential influence by *Genista* shrubs: the area underneath the canopy crown (IN—influenced by litter and roots), and the outer area (OUT) located at least 3 m away from the edge of the nearest shrub canopy, and not previously affected by other individuals of *Genista*. To assess the effect of *Genista* ontogenesis on soil quality, we identified four different developmental stages based on plant age, size, and viability. Shrub age was assessed by standard dendrochronological analysis of 40 randomly selected individuals [46]. The characteristics of the four ontogenetic stages, indicated hereafter as IN_S1_, IN_S2_, IN_S3_, and IN_D_, are (mean and standard deviation of plant age in brackets): IN_S1_ (3.8±0.8 yrs), viable seedlings less than 50 cm tall; IN_S2_ (8.6±1.5 yrs), small shrubs with height ranging between 50 and 200 cm; IN_S3_ (38.4±2.9 yrs) adult plants, isolated reproductive individuals with 3 to 5 stems taller than 200 cm and larger than 10 cm of diameter at ground level; IN_D_ (37.0±1.6 yrs) dead plants with main stems still standing; size similar to IN_S3_. Time since death was not determined.

#### *Genista* fertility island

Soil samples were collected under (IN) and outside (OUT) the canopy of 10 plants of *Genista* randomly selected for each ontogenetic stage, for a total of 80 sampling points (4 stages, 2 areas, 10 randomised replicates). At each sampling point three replicates were collected for a total of 240 soil samples. All samples (~ 2 kg each) were collected from the topsoil layer (0–20 cm), after removing the litter, since such layer includes the rooting depth of most herbaceous plants coexisting with *Genista*. Field survey dates back to 2010 during late spring months (May-June), considered the best time for soil collection [47]. Samples were packed in polyethylene bags, transferred to the laboratory within 3 hours and sieved at 2 mm mesh, with subsequent quantification of the resulting coarse and fine fractions (>2 mm and <2 mm, respectively). Fresh soils were stored at +4°C. Biochemical analysis and bioassay were carried out within 10 days.

Soil texture and chemical properties were assessed on samples dried at room temperature until reaching constant weight. Nineteen variables were measured in order to assess the quality of the fine (< 2 mm) soil fraction. Physical and chemical properties were determined by standard methods [48]. Particle size distribution was determined by the pipette method; pH and electrical conductivity were measured in 1:2.5 soil:water suspensions and 1:5 soil:water extracts, respectively; total carbonates (limestone) were assessed by the Dietrich-Fruehling calcimeter method [49]; organic C content was assayed by chromic acid titration method; total N was determined by flash combustion with a CNS Elemental Analyser (Thermo FlashEA 1112); available phosphate was determined by bicarbonate extraction; cation exchange capacity was measured after soil treatment with a barium chloride and triethanolamine solution at pH 8.2; exchangeable bases (Ca^2+^, Mg^2+^, K^+^, Na^+^) were assayed by flame atomic absorption spectrometry. Microbial activity was assessed by measuring basal respiration and hydrolytic enzymatic activity. The latter was assessed with the Fluorescein Diacetate method (FDA) that measures the amount of enzymatic activity (protease, lipase, non-specific esterase) related to organic matter decomposition (see [50] for method details). Soil respiration was assessed by incubating 50 g of dry soil in screw-cap jars (500 ml). The concentration of CO_2_ in the headspace was measured by the alkali trapping method following Alef [51]. Briefly, 5 ml of 1 N KOH were placed in a test tube inside the screw-cap jar. Alkali was exposed for 48 h with microcosms closed airtight, and then titrated with 0.1 N HCl. Soil hydrophobicity, or water repellency, was measured by the drop infiltration method of air dried soil samples as reported by York and Canaway [52]. Results are reported as concentration and, for C and N, also on a pool basis for a depth of 20 cm.

#### *Genista* litterfall and decomposition

To assess the input of C and N from *Genista* litter, litterfall and litter decomposition in the field were quantified. Litter production was collected by plastic traps (28 cm x 18 cm x 14 cm for each side and height, respectively; walls coated with plastic net with mesh of 1 mm) placed 10 cm above the ground. Litterfall was monthly collected from August 2011 to December 2012, both under the canopy of the randomly selected *Genista* plants, and in the OUT areas, for a total of 80 sampling points (4 ontogenetic stages, 2 sampling areas, 10 randomised replicates). Collected samples were placed in plastic bags and transported to the laboratory, sorted into species, and weighted to dry mass (60°C until constant weight was reached).

The decomposition experiment was carried out in the field, within the IN and OUT areas pertaining to the canopy of adult plants of *Genista* (stage S3, see above), where most litterfall occurs. According to the litterbag method [53], terylene bags (20 x 10 cm^2^, mesh size of 1 mm) were filled with 5 g of dry litter (young branches and leaf), previously collected, air-dried and stored at room temperature (20–25°C), then fixed for incubation on the litter layer by metal pegs. Bags were harvested after 30, 90, 180 and 360 days of decomposition, dried at the laboratory at 40°C until constant weight was reached, and the remaining material weighted afterwards. Decomposition was assessed by measuring litter mass loss. A total of 100 litterbags (1 ontogenetic stages, 2 sampling areas, 10 randomised replicates, 5 decomposition times including undecomposed materials) were analysed.

Total C and N content were determined in undecomposed as well as in decomposed materials by flash combustion of microsamples (5 mg of litter) in an Elemental Analyser NA 1500 (Carlo Erba Strumentazione, Milan, Italy). The content of acid-detergent hydrolysable fibre (thereafter indicated as labile C), proximate cellulose and lignin were assessed following the procedure described by Gessner [54]. Briefly, labile C was determined by mild acid hydrolysis with 0.5 M H_2_SO_4_ added with the detergent cetyltrimethylammonium (CTAB, 20 g l^-1^), proximate cellulose was determined as hydrolysable fraction after drastic sulphuric acid treatment (loss due to 72% H_2_SO_4_ for 3 hours), and proximate lignin as the unhydrolysable fraction (loss upon ignition after the above mentioned H_2_SO_4_ treatment). All carbon fractions are presented as ash-free dry mass.

#### Soil bioassay

A greenhouse bioassay was implemented to assess the effect of soil either unaffected or affected by *Genista* life-cycle on the growth of the same invasive species and of other 5 different coexisting species, typical of the ecological succession in the study area. The experiment was fully factorial, including four levels for the area of soil collection (two IN areas under the canopy of *Genista* individuals from stages S3 and D, and two corresponding OUT areas, respectively) and six levels for the grown plant species. In detail, in addition to *Genista*, we used: i. *Briza maxima* L., an indigenous annual grass widespread on the Vesuvius; ii. *Spartium junceum* L., a native nitrogen-fixing shrub; iii. *Fraxinus ornus* L. subsp. *ornus*, an indigenous mid-successional deciduous tree, and; iv. *Quercus ilex* L. subsp. *ilex*, a native evergreen late-successional tree; v. *Robinia pseudoacacia* L., an invasive nitrogen-fixing tree native to North America that occurs on the Vesuvius Grand Cone.

Soil was collected from 10 randomly selected sampling points in each sampling area. Not sieved soil samples from each area were pooled. Seeds of the 6 target species were collected in the field during the 2010 growing season, from a number of plants > 40, randomly selected for each species. Seeds were placed on Petri dishes for germination at 20°C, with a photoperiod ratio of 16:8 hrs simulating day-night). Pots (14 cm of diameter and 15 cm of height, 15 replicates for each combination of soil source area and grown plant species, for a total of 360 pots) were filled with 500 g of air dried soil and planted with 2 pre-germinated 10 days old seedlings of each target species (1 for *Q*. *ilex* subsp. *ilex*, for space constraints). Pots were placed in a growth chamber (20°C day, 15°C night, natural day-night photoperiod) initially arranged following a random design, then moved with regular rotation. Pots were wetted with distilled water every 2 days, until water holding capacity was reached. After the growing period (220 days for *Q*. *ilex* subsp. *ilex* and 90 days for the other species, respectively) shoots and roots were harvested, washed with tap water, oven dried (60°C until constant weight was reached) and weighted afterwards.

#### *Genista* effects on above- and below-ground microclimate

The majority of previous studies monitored canopy microclimate only for a few days or some weeks [18]. In this study, instead, the monitoring period has been extended for an entire year to assess the differences in microclimatic and soil hydrological regimes respectively underneath and outside the *Genista* canopy.

Abiotic environmental conditions under (IN) and outside the canopy (OUT) of adult *Genista* individuals (stage S3) were characterized by measuring air temperature and relative humidity, as well as soil water content and temperature. We installed two monitoring stations, respectively located at the IN_S3_ and OUT positions, each equipped with a datalogger, two Decagon 5TM integrated moisture content and temperature sensors and an air temperature/humidity sensor (Decagon devices Inc). Air sensor was located at 10 cm above ground with a radiation shield. The two soil moisture and temperature probes were inserted horizontally in the soil, at 5 cm and 20 cm depths, respectively. Moisture sensors have been installed for comparing the rates of temporal changes of moisture content signals IN_S3_ and OUT, as result of the meteorological forcing, rather than for retrieving absolute soil water content values. Thus, no specific direct gravimetric measurements on undisturbed samples were performed for calibrating the relation between 5TM sensor responses and moisture content. Volumetric moisture content data have been retrieved according to the standard relation proposed by the manufacturer, certifying an accuracy ±0.03 cm^-3^cm^3^ for mineral soils that have solution electrical conductivity <10 dS/m, as it occurs in the examined case. The soil temperature sensor employed has an accuracy of ±1°C, with a resolution of 0.1°C. The air temperature probe has an average accuracy of ±0.5°C also with a resolution of 0.1°C. The air relative humidity sensor has a resolution of 0.1% and an average accuracy of 2%. The datalogger was programmed to store sensor data every 10 minutes during the entire monitored year, starting from 16^th^ of January 2012.

To gain further data on the spatial variability of the surface temperature outside and under *Genista* canopy, IR spectra (infrared radiation) of soil surface were acquired by using a thermographic camera (Fluke Ti25), which allowed a non-destructive, rapid and extensive monitoring. Repetitions during bright sunny days in summer 2012 were paired with mid-day measurements of photosynthetic active radiation (PAR, wavelength between 400 and 700 nm) acquired using a light-meter (LI-COR LI-250A) at a height of 0, 50, 100, and 200 cm above the ground, outside and under the canopy of 5 randomly selected individuals of *Genista* for each ontogenetic stage.

#### *Genista* effects on coexisting species

Vegetation surveys were carried out to assess whether *Genista* plants at the four ontogenetic stages were differently associated with coexisting lichens, mosses, herbaceous and woody vascular species, and if plant community structure were different under *Genista* canopy compared with the outer areas. Thus, two main predictive factors were considered for sampling species-species interactions: i. *Genista* ontogenetic stages (S1, S2, S3, D); ii. location with respect to the canopy (IN and OUT areas). For each ontogenetic stage, 10 individuals of *Genista* were randomly selected. Living biomass of coexisting species and species richness of plant community were assessed in both years 2010 and 2011, at the beginning (May) and the end of the growing season (August), by randomly placing three squared sampling frames (20 × 20 cm) within the IN and OUT areas pertaining to each *Genista* individual. The experimental design was fully factorial. Overall, 960 sampling frames were analysed (10 individuals of *Genista* × 4 ontogenetic stages × 2 sampling areas—IN and OUT- × 3 replicated sampling squares × 2 years × 2 dates for year). The above-ground plant biomass within each squared sampling frame was cut with scissors and harvested. The collected material was sorted into dead standing litter and living material of mosses and vascular plants, and kept separated for each species, except for the mosses which were kept aggregated. Vascular plants were identified according to Pignatti [45], Tutin et al. [55,56] and Castroviejo [57]. Nomenclature follows Conti et al. [58,59]. Living biomass and standing litter mass for each species were determined as dry weight, after oven drying at 60°C until constant weight was reached, and expressed as g per m^2^.

#### Data analysis

We tested main and interactive effects of sampling area (IN and OUT) and *Genista* ontogenetic stage (S1, S2, S3, D) on soil properties (texture, organic carbon, total nitrogen, C-to-N ratio, available phosphate, pH, electrical conductivity, cation exchange capacity, exchangeable bases including Ca^2+^, Mg^2+^, K^+^, Na^+^, limestone, hydrolytic enzymatic activity, respiration, and hydrophobicity), above- and below-ground microclimatic variables, and, as well as on vegetation variables (species richness, living biomass both for each recorded species and overall, litterfall of *Genista* and coexisting species. All examined variables were tested for normality and homoscedasticity (by Shapiro-Wilk’s and Levene’s tests, respectively) and transformed when necessary (i.e. Shapiro-Wilk’s test significant at *P*<0.05, S1 Table), using log, arcsin, or square-root transformation functions, to satisfy assumptions required by parametric statistics. Data were submitted to factorial two-way ANOVA for all the above-mentioned variables, with the exception of Photosynthetic Active Radiation. In the latter case, data were submitted to general linear model (GLM) analysis, by adding the effect of height from the ground, treated as a continuous variable, and its interactions with sampling area and ontogenetic stage. Pair-wise significant differences were tested by using post-hoc Duncan test.

Mass loss data from *Genista* litter decomposition experiment were submitted to GLM analysis as well, testing the effects of sampling area (IN and OUT) treated as a fixed factor, decomposition time as a continuous variable, and their interaction. Time-dependent variations of labile C, proximate cellulose and lignin content in litter, as well as total C and N content and C-to-N ratio, were tested for statistical significance by one-way ANOVA.

To assess the effect of soil type on plant shoot and root growth, data from the soil bioassay were submitted to two-way factorial ANOVA. Separate statistical analyses were carried out for each target plant species, considering as treatment factors the ontogenetic stage of the *Genista* individuals (either S3 or D) and the sampling area (either IN or OUT) used as references for soil collection in the field. *Genista* individuals within each stage, seeds within each cultivation pot and pots for each grown plant species were considered randomised experimental replicates, thus not included in the analysed factors.

Vegetation data (species richness, living biomass, and standing litter) were submitted to GLMM (general linear mixed models) analysis. In order to address the environmental heterogeneity associated with the location of *Genista* plants, a randomised block design [60] was used. Mixed models were tested for each species and for all species pooled, with main and second order interactive effects of plant as a random factor, and sampling area (IN and OUT), ontogenetic stage (S1, S2, S3, and D), sampling year (either 2010 or 2011) and season (either May or August) as fixed factors. For a more conservative interpretation of species-level GLMs, the Bonferroni correction was adopted to calculate critical *p*-values for significance (*p* = 0.00125), by dividing 0.05 by the number of species considered in the analysis (*N* = 40). Pair-wise differences among ontogenetic stages within each sampling area, and between IN and OUT means within each ontogenetic stage were tested for significance by using the post-hoc *t* test. In the case of ontogenetic stages, the Bonferroni correction was applied to post-hoc *t* results. The critical *p*-value for statistical significance (*p* = 0.00834) was obtained dividing 0.05 by the total number *n* of pair-wise comparisons [i.e. *n* = *k* (*k*-1)/2 = 6, with *k* = 4 for the number of ontogenetic stages]. Such correction was not needed in the case of sampling area (*n* = 1, with k = 2). All statistics were calculated using the software package STATISTICA 7 (Stat-Soft Inc., USA); all statistical results were considered significant at *p*<0.05, unless otherwise specified.

## Results

### *Genista* fertility island

All soil samples showed a sandy texture, with slight variations of sand, silt and clay irrespective of the presence and age of *Genista* canopy (Tables 1 and S2).

**Table 1:** Soil variables under the canopy of *Genista aetnensis*.

Variable	IN				OUT
	S1	S2	S3	D	(all stages)
Texture					
Fraction >2 mm (mg g^-1^)	772±31 a	728±22 a	784±27 a	735±30 a	766±6
Fraction <2 mm					
Sand (mg g^-1^)	967±5 a	957±6 a	961±11 a	979±8 a	963±3
Silt (mg g^-1^)	11±5 a	9±5 a	17±7 a	14±9 a	18±3
Clay (mg g^-1^)	22±6 a	34±16 a	22±9 a	7±6 a	17±3
Organic C (mg g^-1^)	3.32±0.03 a	8.71±2.25 a	45.03±9.25 b	49.37±5.97 b	4.43±0.54 ***
Total N (mg g^-1^)	0.34±0.03 a	0.74±0.14 a	4.44±0.87 b	4.95±0.98 b	0.46±0.05 ***
C-to-N ratio	9.81±0.85 a	11.34±1.46 a	10.14±0.33 a	10.29±0.89 a	10.40±0.43
P_2_O_5_ (mg kg^-1^)	5.93±0.09 a	14.75±2.30 a	53.43±11.09 b	56.28±10.08 b	8.08±1.07 ***
pH	6.39±0.08 b	6.06±0.11 b	5.42±0.12 a	5.40±0.16 a	6.48±0.05 ***
Electrical conductivity (dS m^-1^)	0.042±0.001 a	0.123±0.003 b	0.701±0.146 c	0.404±0.109 bc	0.050±0.003 ***
CEC (meq+ 100 g^-1^)	2.02±0.12 a	2.69±0.34 a	13.62±4.60 b	16.33±2.06 b	2.19±0.10 ***
K^+^ (meq+ 100 g^-1^)	0.18±0.01 a	0.30±0.02 a	0.52±0.07 b	0.49±0.07 b	0.21±0.01 ***
Mg^2+^ (meq+ 100 g^-1^)	0.22±0.02 a	0.27±0.02 a	1.68±0.61 b	1.14±0.20 b	0.19±0.01 ***
Ca^2+^ (meq+ 100 g^-1^)	1.42±0.08 a	1.74±0.31 a	10.61±3.65 b	12.53±1.11 b	1.49±0.10 ***
Na^+^ (meq+ 100 g^-1^)	0.10±0.01 a	0.10±0.01 a	0.12±0.01 a	0.11±0.02 a	0.12±0.01
Limestone	0.50±0.01 a	5.32±1.59 ab	6.72±3.03 ab	8.39±2.11 b	2.49±0.75 *
FDA (μg g^-1^ h^-1^)	197±43 a	250±35 a	286±44 a	306±96 a	130±61 *
Respiration (μg CO_2_-C g^-1^ soil h^-1^)	1.25±0.24 a	1.81±0.37 ab	3.89±0.28 c	2.78±0.37 b	1.32±0.09 ***
Hydrophobicity (s)	<1±0 a	1248±1177 a	12033±843 c	7760±288 b	<1±0 ***

Properties of topsoil (0–20 cm) in the survey area under the canopy (IN) of *Genista aetnensis* individuals at four ontogenetic stages (S1, S2, S3, D). For each variable, data refer to mean ± s.e.m. of 10 replicates. Different letters indicate statistically significant differences among ontogenetic stages within the IN area (post-hoc Duncan test; *P*<0.05). For comparison, data for the area outside the canopy (OUT) are also reported, pooled for all ontogenetic stages, with indication of significant differences between the two sampled areas (post-hoc Duncan test; *: *p*<0.05; **: *p*<0.01; ***: *p*<0.001). Detailed statistical results are in S1–S3 Tables.

Conversely, significant differences of organic C stock, total N, extractable P, pH, electrical conductivity, cation exchange capacity, K^+^, Mg^2+^, Ca^2+^, limestone content, and microbial activity assessed both as respiration and FDA enzymatic activity were found between the areas under (IN) and outside (OUT) the canopy of *Genista* (Table 1, S2 Table). For most soil variables a significant effect of *Genista* ontogenetic stage was found within the IN area (Table 1), but not within the OUT area (S3 Table).

In detail, organic C stock in the upper soil layer increased from 3.32±0.03 g_C_ kg^-1^_soil_ under the canopy of *Genista* seedlings (IN_S1_) to significantly higher values of 45.0±9.3 g_C_ kg^-1^_soil_ and 49.4±6.0 g_C_ kg^-1^_soil_ under adult living (IN_S3_) and dead (IN_D_) plants, respectively (Table 1), being more than ten times higher compared with the OUT area (4.4±0.5 g_C_ kg^-1^_soil_, Table 1, S3 Table). The same pattern was observed for total N stock, increasing from 0.34±0.03 g_N_ kg^-1^_soil_ (IN_S1_) and 0.46±0.05 g_N_ kg^-1^_soil_ (OUT) to 4.44±0.87 g_N_ kg^-1^_soil_ (IN_S3_) and 4.95±0.98 g_N_ kg^-1^_soil_ (IN_D_) in ~ 40 years of colonization (Table 1). Interestingly, C-to-N ratio did not vary significantly, neither between the areas under and outside the canopy of *Genista*, nor among individuals at different ontogenetic stage (Table 1, S2 Table) indicating that the increase rates of soil organic C and total N during the *Genista* ontogenesis are not significantly different. A progressive, significant increase with *Genista* ontogenetic stage was also found for below-canopy soil content of available P, acidity, electrical conductivity, CEC, exchangeable bases, with the only exception of Na^+^ content (Table 1, S2 Table). Soil microbial activity under *Genista* canopy showed a similar trend, with averages of respiration and FDA increasing with *Genista* ontogenetic stage. However, in the case of FDA the effect was not statistically significant, due to a large variability among the replicates (Table 1). Soil in the OUT areas, as well as under *Genista* seedlings, was not hydrophobic, with drops immediately penetrating soil surface. In contrast, soil hydrophobicity in the IN area progressively increased with the ontogenetic development *Genista* individuals, with drops persisting over the soil surface for even more than 3 h, as in the case of soil under dead adults (Table 1).

### *Genista* litterfall and decomposition

As expected, *Genista* litterfall was very low outside the canopy (OUT area, 29±15 g m^-2^ yr^-1^) and much higher under the canopy (IN area, 509 ±58 g m^-2^ yr^-1^) of *Genista* individuals. However, litterfall largely varied according to the ontogenetic stage of the plant, being lower in the cases of seedlings (IN_S1_, 213±49 g m^-2^ yr^-1^) and dead adults (IN_D_, 270±72 g m^-2^ yr^-1^), and progressively increasing under the canopy of juveniles (IN_S2_, 554±80 g m^-2^ yr^-1^) and living adults (997±44 g m^-2^ yr^-1^). Differences between the two sampling areas and among ontogenetic stages within the IN area were both statistically significant (two-way ANOVA, F_1,72_ = 231.4, *p*<0.0001, F_3,72_ = 33.2, *p*<0.0001, F_3,72_ = 31.4, *p*<0.0001 for the effects of sampling area, ontogenetic stage, and the interaction term, respectively, S4 Table).

Litterfall of other plant species was much lower compared with *Genista* litterfall. Differences between values recorded under and outside the *Genista* canopy (78±34 g m^-2^ yr^-1^ and 23±5 g m^-2^ yr^-1^ for the IN and OUT areas, respectively) were statistically significant (F_1,72_ = 12.7, *p* = 0.0006 for the effect of sampling area, S4 Table) as well as differences recorded under the canopy of individuals at different ontogenetic stages (F_3,72_ = 3.84, *p* = 0.0130 for the effect of ontogenetic stage, S4 Table). Litterfall by heterospecifics found under dead *Genista* adults (IN_D_, 162±55 g m^-2^ yr^-1^) were higher compared to living adults (IN_S3_, 79±21 g m^-2^ yr^-1^), juveniles (IN_S2_, 39±13 g m^-2^ yr^-1^), and seedlings (IN_S1_, 31±5 g m^-2^ yr^-1^).

*Genista* seed dispersal, although highly variable among the sampling sites, was significantly clumped with the seed source, with 309±154 seeds m^-2^ yr^-1^ and 13±3 seeds m^-2^ yr^-1^ in the IN and OUT areas, respectively (F_1,72_ = 17.4, *p*<0.0001, S4 Table). Considering the area under the canopy, seed abundance progressively increased with ontogenetic stage of the source plant, with 512±226 seeds m^-2^ yr^-1^, 162±61 seeds m^-2^ yr^-1^ and 18±11 seeds m^-2^ yr^-1^ for *Genista* adult plants (IN_S3_), juveniles (IN_S2_) and seedlings (IN_S1_), respectively.

**Fig 2:**
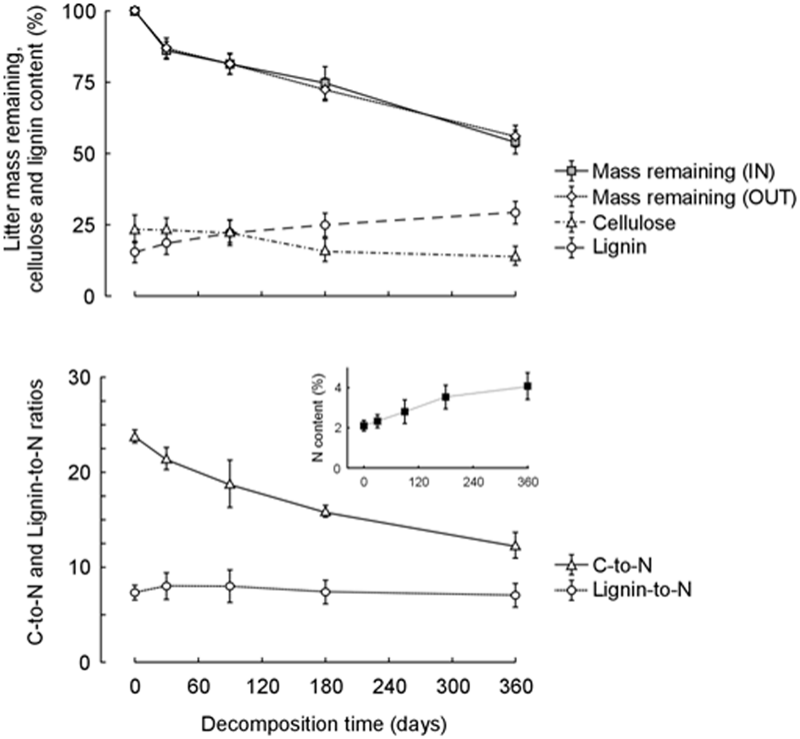
Results of the litter decomposition experiment. Dynamics of litter mass (% of initial value) in litterbags decomposing for 360 days under (IN) and outside (OUT) the canopy of adult *Genista aetnensis* individuals, and corresponding changes of cellulose and lignin percent content (top), C-to-N and lignin-to-N ratios (bottom) and N percent content (inset). Data refer to mean and standard deviation of 10 replicates for each harvesting date. Data of chemical variables (cellulose, lignin, N, C-to-N and lignin-to-N ratios) are pooled for IN and OUT areas, since such factor did not affect significantly litter decomposition in the field (S6 Table).

Litter decomposition did not show significant variations between litterbags placed under and outside *Genista* canopy (Fig 2A, S5 Table). On the contrary, all litter variables significantly changed during decomposition time (S6 Table).

Mass loss was initially rapid (from 5±0.001 g of undecomposed materials to 4.33±0.02 g and 4.37±0.02 g after 30 days in the IN and OUT areas, respectively) then slowed between 30 and 180 days (3.79±0.05 g and 3.67±0.02 g after 180 days in the two areas), but a significant mass loss was still observed between 180 and 360 days of decomposition (Fig 2A, S6 Table). Overall, *Genista* litter lost 45% of its initial mass during one year of decomposition. Proximate cellulose and lignin content in undecomposed *Genista* litter were relatively high (23.57±0.28% and 15.45±0.21%, respectively). Both variables significantly varied during decomposition (S6 Table). Cellulose started to decrease only after 90 days, with a total loss of 39.9%; lignin considerably increased throughout the decomposition period, with an overall rise of 89.5% (Fig 2A).

The concentration of N in the litter residuals significantly increased during the 360 days of decomposition (S6 Table). In this period the average concentration of N increased of 98%, from 2.11±0.01% of undecomposed litter up to 4.17±0.08 after 360 days (Fig 2B). The C-to-N ratio followed an exponential decrease during the decay process, with a significant average value of 48.2% reduction, from 23.8±0.1 to 12.3±0.2 (Fig 2B, S6 Table). Interestingly, lignin-to-N ratio followed a peculiar dynamic. First, it slightly increased from the value of 7.3±0.1 of undecomposed litter to 8.0±0.2 and 9.0±0.3 after 30 and 90 days of decomposition, respectively. Then, it decreased down to values not significantly different from the initial (7.4±0.2 and 7.1±0.2 after 180 and 360 days, respectively).

### Soil bioassay

**Fig 3:**
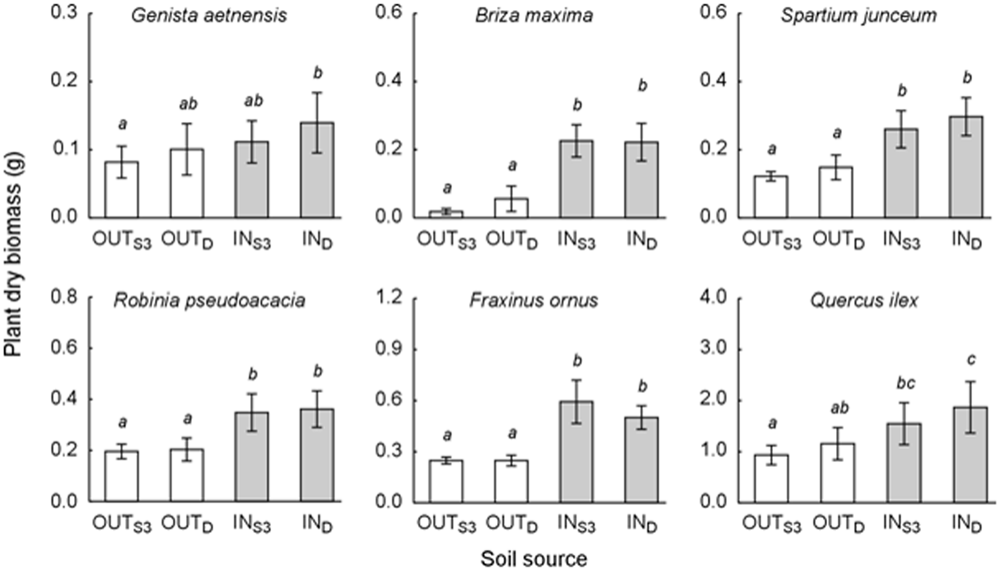
Plant growth in the greenhouse bioassay. Plant dry biomass of six target species pot-grown on soil collected at the Vesuvius Grand Cone, outside and under the canopy of either living (OUT_S3_ and IN_S3_, respectively) or dead (OUT_D_ and IN_D_, respectively) *Genista aetnensis* adult individuals. For each species and within each treatment, data refer to mean and 95% confidence interval of single plant dry mass (g) from 15 replicated pots. Different small letters indicate statistically significant difference among soil types (Post-hoc Duncan test at *p*<0.05, from two-way ANOVA in S7 Table).

In the bioassay, most of the target species showed significantly higher biomass when grown on soil collected under the canopy of *Genista* individuals, than on soil from the area outside the canopy, irrespective of the *Genista* ontogenetic stage (Fig 3, S7 Table).

The annual grass *Briza maxima* showed the most relevant biomass increase when exposed to soil affected by living (IN_S3_) and dead (IN_D_) *Genista* adults, with +1143% and +255% compared to corresponding unaffected soil types from the OUT area (Fig 3). Accordingly, *Fraxinus ornus* subsp. *ornus*, *Spartium junceum*, *Robinia pseudoacacia*, and *Quercus ilex* subsp. *ilex* showed a common pattern, though with a progressively lower magnitude of the soil type effect. In detail, biomass increases when passing from soil unaffected by *Genista* (OUT) to affected soils (IN) were +139% (IN_S3_) and +101% (IN_D_) in the case of *F*. *ornus* subsp. *ornus*, + 113% (IN_S3_) and +100% (IN_D_) for *S*. *junceum*, +77.8% (IN_S3_) and +77.7% (IN_D_) for *R*. *pseudoacacia*, and +65.7 (IN_S3_) and +45.4 (IN_D_) in the case of *Q*. *ilex* subsp. *ilex* (Fig 3). For this latter species, seedling growth on soil collected under and outside the canopy of dead *Genista* plants (IN_D_) was considerably higher than on corresponding soils from living adults (IN_S3_) (Fig 3). Such differences were not statistically significant, even if the associated *p*-value was very close to the limit *p*-value pf 0.05 (S7 Table). Similarly, no significant interactive effects were found between the two factors considered for characterising the soil type. Interestingly, the growth of *Genista* seedlings was much less affected by the soil type compared with the other species (Fig 3). The general effect of the sampling area (IN *vs* OUT) was still statistically significant (S7 Table), whereas the effect of the ontogenetic stage was not, as in the case of all other target species. However, as pointed out by post-hoc statistics, *Genista* seedlings showed non-significantly different growth on soils, either affected or unaffected by conspecifics at the same ontogenetic stage (Fig 3).

### *Genista* effects on microclimate and soil hydrological regime

**Fig 4:**
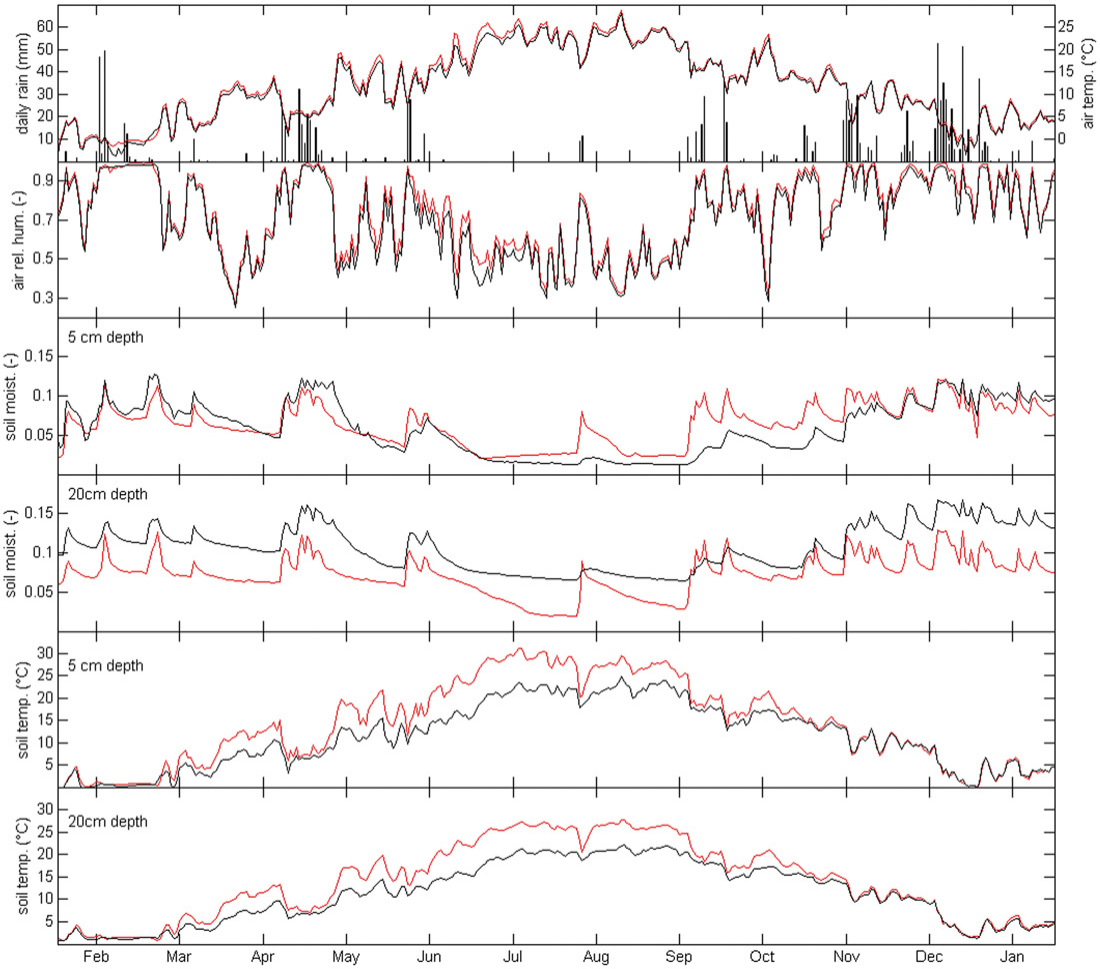
Time series of microclimate variables. Daily values of cumulated rainfall, average air temperature and relative humidity, soil temperature and water content measured at both 5 cm and 20 cm depths outside (OUT—red lines) and under the canopy of living (IN_S3_—black lines) *Genista aetnensis* adult individuals. Data refer to 1 year of monitoring (January 2012 – January 2013).

Microclimate (rainfall, average air temperature and relative humidity) and soil state variables (water content measured at both 5 cm and 20 cm depths) exhibit a strong seasonality, typical of the Mediterranean regions (Fig 4).

From a soil hydrological perspective, four stages can be distinguished during the monitored year: a wet stage from January to April and from November to December; a wet-to-dry transition stage from May to June; a dry stage from July to August; a dry-to-wet transition stage from September to October (Fig 4). In the wet stage, soil moisture time series exhibit a sequence of pulses corresponding to rainfall events and values stably above a threshold value which can be assimilated to field capacity conditions [61]. This temporal pattern is typically observed in temperate regions when precipitation largely exceeds the evapotranspiration losses [62]. In the first half of February the field was covered by a snow layer up to 60 cm thick (Chirico G.B., personal observation), thus submerging completely the air relative humidity and temperature sensors. As a result, both soil and air temperatures probes recorded almost constant values, with air temperature close to 0°C, soil temperature around 0.8°C at 5 cm depth and 1.6°C at 20 cm depth, while air relative humidity close to 100%. In the dry stage soil water content is almost constant at minimum values, except for an isolated peak corresponding to a rainfall burst typical of the summer seasons in Mediterranean regions [63]. In dry-to-wet transition, the soil moisture patterns IN and OUT are markedly different. The soil moisture time series outside the canopy (OUT) are characterised by sharp peaks in response to the rainfall events and the soil state rapidly switches from the dry stage to the wet stage after the rainfall events of September (Fig 4). On the contrary, the soil under the canopy (IN) exhibits a milder response to the rainfall during this period, with a much slower transition from the dry stage to the wet stage, which is reached only in November (Fig 4). The effect of canopy cover is particularly evident on the temperature regimes during the dry-to-wet and dry stages. With respect to outside the canopy, the mean air temperature underneath the canopy is reduced by 0.6°C during the entire year, and by 1°C in the period May-August. The effect is even more pronounced on the soil temperature regimes. The mean soil temperature under the canopy (IN) from May to August, is on average 5.8°C lower at 5 cm depth and 5.3°C lower at 20 cm depth, compared with what observed outside the canopy (OUT).

The greatest differences between IN and OUT tend to occur in the hottest time of the day (S1 Fig). In detail, from May to August, the 90^th^ percentile (p_90_) of the temperature difference between IN and OUT was 1.8°C above ground, 8.4°C at 5 cm depth and 5.8°C at 20 cm depth. While outside the canopy the surface soil temperature often reaches values larger than 30°C during the hottest hours of the dry stage (33.3°C at p_90_), underneath the canopy the surface soil temperature was almost always below 25°C (24.6°C at p_90_). The daily range of the surface soil temperature was also mitigated underneath the canopy. The difference between the 90^th^ and the 10^th^ percentiles was 4.8°C smaller underneath the canopy than outside (S2 Fig). These differences in soil temperature are expected to dramatically increase moving closer to the soil surface. Infrared images taken in a sunny, summer day (13 August 2012), revealed surface temperature often higher than 60°C on the bare soil, with highest temperatures recorded at OUT surface reaching the astonishing value of +81°C, that contrast with air and soil surface temperature below the *Genista* canopy that was around 28°C and 30°C, respectively (S3 Fig). The air relative humidity was generally higher under the canopy than outside, especially in the period from May to August (Figs 4, S1 and S2), when the average difference is around 5%. The difference in relative humidity between OUT and IN was negatively correlated with the corresponding air temperature difference, with a linear correlation coefficient equal to -0.86 (S4 Fig), i.e. for a decrease in air temperature of 4°C the average increase of air relative humidity is 15%.

**Fig 5:**
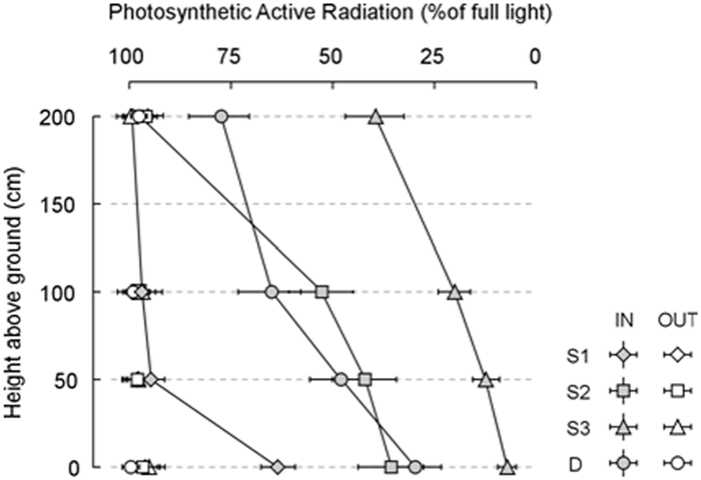
Effects of *Genista aetnensis* canopy on light regime. Photosynthetic active radiation (PAR) attenuation along the vertical profile under (IN) and outside (OUT) the canopy of *Genista aetnensis* individuals at different ontogenetic stage (S1, S2, S3, D) at the Vesuvius Grand Cone. Data refer to mean and s.e.m. of PAR, expressed as percentage of the maximum recorded value (full light, 1171 μmol m^2^ s^-1^). See S8 Table for statistical analysis.

Significant PAR attenuation occurred along the vertical profile under the canopy of *Genista* individuals, but not outside the canopy, where full light conditions (mean and s.e.m. 1115±28 μmol m^-2^s^-1^) were always recorded (Fig 5).

Considering *Genista* ontogenetic stage, PAR attenuation was particularly evident under adults (IN_S3_, from 464±72 μmol m^-2^s^-1^ at 2 m above-ground to 84±11 μmol m^-2^s^-1^ at ground level), and to a less extent under juveniles (IN_S2_, from 619±80 μmol m^-2^s^-1^ at 1 m to 417±81 μmol m^-2^s^-1^). Under dead adults (IN_D_), notwithstanding their stem size and height, mid-light condition were recorded due to canopy shading (from 913±100 μmol m^-2^s^-1^ at 1 m to 353±85 μmol m^-2^s^-1^), whereas under *Genista* seedlings (IN_S1_), at a height of 50 cm above-ground, PAR was still rather high (742±21 μmol m^-2^s^-1^). As expected, PAR did not vary significantly along the vertical profile outside and above the canopies (Fig 5). Accordingly, a significant effect on PAR intensity was found for sampling area (IN *vs* OUT), ontogenetic stage, height from the ground, and for the interaction terms (S8 Table).

### *Genista* effects on coexisting species

**Fig 6:**
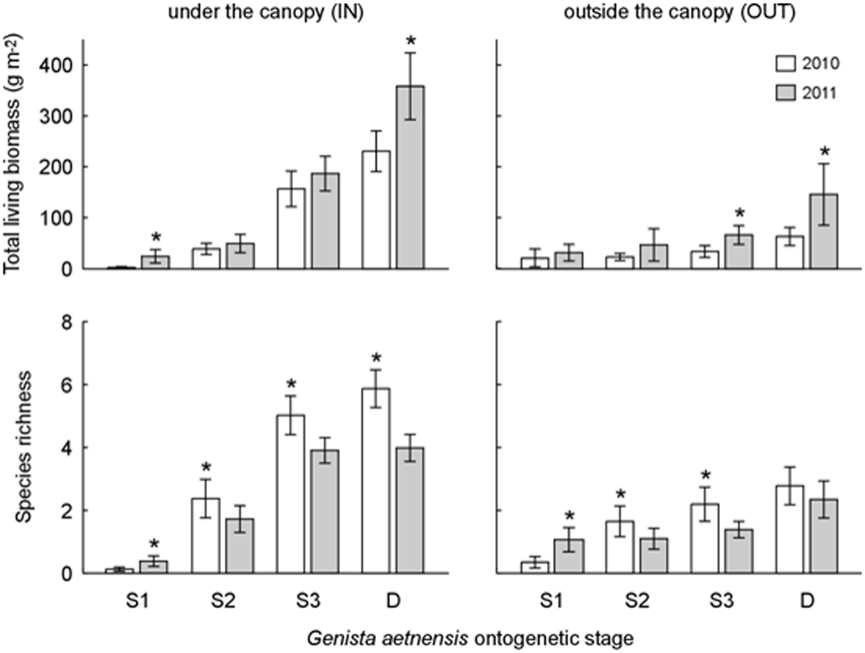
Yearly effects of *Genista aetnensis* on vegetation. Time-dependency of the effects of *Genista aetnensis* ontogenetic stage on plant living biomass (top) and species richness (bottom) under (left) or outside (right) the canopy of *Genista* individuals. Data refer to mean and 95% confidence interval of data recorded in two surveys in the years 2010 and 2011 (N = 60 for each bar). Significantly higher time-dependent values within each ontogenetic stage are marked with an asterisk (Post-hoc Duncan test at *p*<0.05, interactions of year and ontogenetic stage from GLMs in S10 Table).

Overall, 68 taxa were recorded at the study sites (S9 Table). Most plants are either annual or perennial grasses, but seedlings of shrubs and trees were also found, as well as one lichen (*Stereocaulon vesuvianum* Pers.) and several mosses (S9 Table). Plant diversity was far more high under *Genista* canopies compared to the outside areas, as coherently showed by distributional patterns (Table 2, Fig 6) of total living biomass (mean and s.e.m. of 216.1±12.3 g m^-2^ and 50.5±4.6 g m^-2^ under and outside the canopy, respectively), standing litter (540.2±26.0 g m^-2^ and 1.6±0.5 g m^-2^), and species richness (3.18±0.13 and 1.48±0.09 species per plot, respectively).

**Table 2:** Effects of *Genista aetnensis* on coexisting species.

Taxon	IN				OUT			
	S1	S2	S3	D	S1	S2	S3	D
**Lichens and mosses**								
*Stereocaulon vesuvianum*	-	-	-	0.36±0.28 *a*	0.36±0.34 *a*	1.7±0.9 *a*	0.92±0.74 *a*	1.23±0.79 *a*
Mosses (pooled data)	0.02±0.02 *a*	1.22±1.01 *a*	**22.1±7.5** *b*	**86.29±10.81** *c*	-	-	0.08±0.07 *a*	1.33±1.21 *a*
**Herbaceous annuals**								
*Aira caryophyllea subsp*. *caryophyllea*	-	0.05±0.04 *a*	0.01±0.01 *a*	0.01±0.01 *a*	-	0.01±0.01 *a*	0.77±0.61 *a*	0.42±0.33 *a*
*Avena barbata*	-	0.70±0.45 *a*	9.29±2.70 *a*	**28.33±7.48** *b*	-	-	0.07±0.07 *a*	2.22±1.10 *a*
*Briza maxima*	-	11.96±3.97 *a*	**96.25±8.91** *c*	**68.81±8.82** *b*	-	1.28±0.40 *a*	3.18±0.94 *a*	9.16±2.76 *a*
*Bromus sterilis*	-	-	**12.82±5.05** *b*	**16.94±4.93** *b*	-	0.02±0.02 *a*	-	0.08±0.08 *a*
*Bromus tectorum subsp*. *tectorum*	-	1.19±0.42 *a*	1.02±0.34 *a*	0.50±0.31 *a*	0.26±0.13 a	1.04±0.33 *ab*	1.75±0.52 *ab*	**2.83±1.13** *b*
*Carduus pycnocephalus subsp*. *pycnocephalus*	-	0.41±0.32 *a*	**2.81±0.20** *b*	0.44±0.30 *a*	-	-	0.15±0.14 *a*	-
*Cynosurus echinatus*	-	-	**10.2±4.46** *b*	2.25±1.23 *a*	-	-	-	-
*Galium aparine*	-	0.13±0.08 *a*	**3.53±1.4** *b*	2.2±0.53 *ab*	-	0.06±0.04 *a*	0.16±0.14 *a*	-
*Geranium purpureum*	-	0.06±0.05 *a*	**4.14±1.38** *b*	**7.25±2.16** *b*	-	-	0.4±0.18 *a*	1.22±0.50 *a*
*Lactuca serriola*	-	-	2.42±2.32 *a*	**22.34±7.67** *b*	-	-	-	-
*Myosotis arvensis* subsp. *arvensis*	-	-	0.02±0.02 *a*	**2.05±0.83** *b*	-	0.14±0.14 *a*	0.12±0.07 *a*	0.28±0.15 *a*
*Sonchus asper* subsp. *asper*	-	-	-	0.07±0.06 *a*	-	-	-	-
*Trifolium arvense* subsp. *arvense*	0.24±0.15 *a*	3.77±1.07 *b*	2.28±0.96 *b*	-	2.35±0.83 *a*	5.54±1.38 *a*	3.19±0.87 *a*	4.07±2.35 *a*
*Vulpia myuros*	-	1.87±0.69 *a*	**7.94±2.36** *b*	0.53±0.35 *a*	0.13±0.09 *a*	0.81±0.31 *a*	1.66±0.99 *a*	2.09±0.78 *a*
**Herbaceous perennials**								
*Arabis collina* subsp. *collina*	-	5.67±3.86 a*b*	**21.94±7.59** *c*	5.49±2.46 *b*	1.16±1.16 *a*	0.03±0.03 *a*	1.01±0.86 *a*	5.98±2.91 *b*
*Arabis turrita*	-	-	0.22±0.14 *a*	**16.36±6.18** *b*	-	-	0.01±0.01 *a*	0.63±0.45 *a*
*Arrhenatherum elatius* subsp. *elatius*	-	-	**27.04±8.23** *b*	**98.34±13.41** *c*	-	-	4.4±1.95 *a*	4.33±2.42 *a*
*Artemisia campestris* subsp. *variabilis*	-	-	0.33±0.24 *a*	-	1.04±0.76 *a*	3.12±1.71 *a*	0.11±0.11 *a*	-
*Centaurea deusta*	-	1.07±1.07 *a*	-	-	-	-	-	0.81±0.78 *a*
*Centranthus ruber* subsp. *ruber*	-	0.05±0.04 *a*	2.7±1.98 *a*	**32.66±11.52** *b*	3.6±1.79 *a*	3.8±2.4 *a*	0.53±0.38 *a*	5.72±3.22 *a*
*Dactylis glomerata* subsp. *glomerata*	-	-	-	2.2±2.2 *a*	-	-	-	-
*Daucus carota* subsp. *carota*	-	3.27±1.74 *a*	**7.07±1.27** *b*	**24.68±4.5** *c*	-	0.48±0.48 *a*	0.16±0.14 *a*	4.93±3.71 *a*
*Glaucium flavum*	-	-	-	0.51±0.51 *a*	-	1.39±1.39 *a*	2.41±1.57 *a*	4.43±3.22 *a*
*Hieracium piloselloides*	-	1.75±1.26 *a*	1.84±0.98 *a*	3.86±1.94 *a*	-	-	1.01±0.78 *a*	**15.05±4.11** *b*
*Hypochaeris radicata*	-	-	0.55±0.39 *a*	2.56±1.99 *a*	4.06±3.26 *a*	7.19±5.69 *a*	2.76±2.43 *a*	-
*Lactuca muralis*	-	-	-	1.03±0.92 *a*	-	-	-	-
*Linaria purpurea*	-	1.66±1.39 *a*	1.05±0.76 *a*	1.26±0.94 *a*	-	-	-	-
*Petrorhagia dubia*	-	0.53±0.32 *a*	1.05±0.43 *a*	-	0.42±0.38 *a*	0.62±0.49 *a*	0.74±0.39 *a*	-
*Picris hieracioides* subsp. *spinulosa*	0.24±0.21 *a*	4.15±2.71 *a*	5.05±3.16 *a*	8.07±4.36 *a*	2.71±2.71 *ab*	1.18±0.91 *ab*	0.52±0.46 *a*	10.93±7.09 *b*
*Rumex acetosella* subsp. *angiocarpus*	0.03±0.03 *a*	1.63±0.98 *a*	3.04±2.67 *a*	-	-	0.84±0.68 *a*	1.82±0.88 *a*	2.99±1.62 *a*
*Rumex scutatus* subsp. *scutatus*	0.004±0.003 *a*	5.54±2.11 *a*	**54.1±8.78** *c*	21.25±8.11 *b*	4.17±2.14 *a*	3.3±1.29 *a*	13.34±3.86 *a*	12.61±4.85 *a*
*Scrophularia canina* subsp. *bicolor*	-	-	-	-	3.41±3.1 *a*	0.83±0.63 *a*	3.74±2.48 *a*	0.58±0.58 *a*
*Silene vulgaris* subsp. *tenoreana*	-	0.73±0.44 *a*	**5.71±1.99** *b*	-	1.45±1.15 *a*	-	-	-
*Solidago virgaurea* subsp. *virgaurea*	-	3.77±3.07 *a*	-	-	-	-	-	-
**Vines, shrub & trees**								
*Clematis vitalba*	-	-	4.29±2.84 *a*	**14.61±4.06** *b*	-	-	-	-
*Cytisus scoparius* subsp. *scoparius*	-	-	0.56±0.46 *a*	4.51±2.75 *a*	-	-	2.66±1.76 *a*	6.33±4.53 *a*
*Pinus nigra* subsp. *nigra*	-	-	-	-	-	-	0.04±0.03 *a*	-
*Robinia pseudoacacia*	-	-	-	1.05±0.74 *a*	-	-	-	1.14±1.01 *a*
**Total living biomass**	0.5±0.2 *a*	52.9±6.8 *b*	**293.5±17.0** *c*	**396.9±23.9** *d*	25.1±6.6 *a*	34.4±8.1 *ab*	49.5±5.6 *ab*	104.1±16.2 *b*
**Total standing litter**	9.6±2.5 *a*	**315.3±25.1** *b*	**990.3±41.3** *d*	**589.6±40.9** *c*	0.36±0.27 *a*	0.17±0.09 *a*	1.45±0.78 *a*	5.59±2.08 *a*
**Species richness**	0.11±0.05 *a*	1.95±0.19 *b*	**4.37±0.19** *c*	**4.83±0.2** *c*	0.61±0.11 *a*	1.28±0.15 *ab*	1.69±0.15 *bc*	2.39±0.20 *c*

Plant species, lichens and mosses distribution under (IN) and outside (OUT) the canopy of *Genista aetnensis* individuals at four different ontogenetic stages (S1, S2, S3, D). Data refer to mean and s.e.m. of living biomass (g m^-2^) of the 40 most recorded species, total living biomass (g m^-2^), total standing litter (g m^-2^), and species richness, recorded in squared plots of 20 × 20 cm^2^ (*N* = 30 for each level of the factorial design) and pooled for four sampling dates (May and August of years 2010 and 2011). For each table row, different small letters and values in bold indicate significant differences dependent on *Genista* ontogenetic stage within each sampling area, and significantly higher values within each ontogenetic stage, respectively (post-hoc *t* test Bonferroni’s correction from GLMs in S10 and S11 Tables, *p*<0.00834 and *p*<0.05, respectively). Zero values and corresponding letters (*a*) are omitted to improve readability.

For all these variables, differences were statistically significant not only between the IN and OUT areas overall, but also considering pair-wise differences within each ontogenetic stage of *Genista* individuals (Tables 2 and S9).

Interestingly, the general enhancing effect of *Genista* on plant diversity progressively increased with its ontogenetic stage. Biomass, standing litter, and species richness under the canopy of adult plants were 5.5, 3.1, and 2.2 times higher than under juveniles, respectively, and corresponding values under seedlings were even lower, by at least one order of magnitude (Table 2). Plant diversity under the canopy of dead *Genista* plants was considerably high, though the three considered variables followed a different pattern: biomass and standing litter were significantly higher (135.2%) and lower (59.5%) than under adult plants, while differences of species richness were not significant (Table 2). Plant diversity in the areas outside the canopy was much less affected by the ontogenetic stage of the closest *Genista* individual, as indicated by the significant interaction term in the GLMM models of biomass, standing litter, and species richness (S10 Table). The general increasing trend observed under the canopy here was still detectable, but generally statistically non significant (Table 2). Exceptions were a lower plant biomass outside *Genista* seedlings, compared to adults, and a higher species richness outside the canopy of adult plants, both living and dead, compared to juveniles and seedlings (Table 2).

According to GLM models for plant diversity measures, variations over time of total plant biomass and species richness were statistically significant. In the case of biomass, a significant effect of the year of survey, but not of the season, was found, whereas species richness significantly varied both yearly and seasonally. On the contrary, standing litter did not change significantly over time. Considering interactive effects, only 3 time-dependent effects out of 12 tested cases were statistically significant. These were year × ontogenetic stage for biomass and species richness, and year × sampling area for species richness (S10 Table). However, time-dependent variations did not affect the general trend of increasing plant diversity under older *Genista* canopy, which was systematically observed in both years 2010 and 2011 (Fig 6).

Considering the biomass distribution of the 40 most frequent species, in the majority of cases plants were unevenly distributed under and outside *Genista* canopies, selectively or preferentially occurring under older individuals (Tables 2 and S11). In detail, 8 plant species were exclusively found, and 17 taxa were most abundant, under *Genista* canopies (S12 Table). Biomass differences between the IN and OUT areas, when data were pooled for all *Genista* individuals, were statistically significant for 6 species (*Briza maxima* L., *Arrhenatherum elatius* [L.] P. Beauv. ex J. et C. Presl subsp. *elatius*, *Avena barbata* L., *Bromus sterilis* L., *Galium aparine* L., and *Geranium purpureum* Vill.), all among the most frequently observed plants. When data from *Genista* individuals at different ontogenetic stage were considered separately, 18 plant species resulted significantly associated with living and/or dead adults (Table 2). These included several herbaceous species (e.g. *Arabis collina* Ten. subsp. *collina*, *Briza maxima* L., *Cynosurus echinatus* L., *Rumex scutatus* L. subsp. *scutatus*, and *Vulpia myuros* [L.] C.C. Gmel.) significantly most abundant under living plants, others most commonly found under dead plants (e.g. *Arabis turrita* L., *Arrhenatherum elatius* [L.] P. Beauv. ex J. et C. Presl subsp. *elatius*, *Avena barbata* L., *Centranthus ruber* [L.] DC. subsp. *ruber*, *Daucus carota* L. subsp. *carota*, and *Lactuca serriola* L.), and two species (i.e. *Bromus sterilis* L., and *Geranium purpureum* Vill.) with not significantly different biomass in the two areas. The vine *Clematis vitalba* L. and the native shrub *Cytisus scoparius* (L.) Link subsp. *scoparius* occurred preferentially under dead *Genista* individuals. On the contrary, no species were preferentially distributed under the canopy of younger *Genista* individuals (Table 2). The biomass of all mosses was significantly higher under *Genista* canopy, both considering data pooled from all ontogenetic stages, and separately from living and dead adults (Tables 2 and S12). In these latter cases, biomass under the canopy were 276 and 65 times higher than the corresponding records from outer areas.

Only few species (e.g. the early colonizer lichen *Stereocaulon vesuvianum* Pers., the herbaceous annual *Trifolium arvense* L. subsp. *arvense*, and the perennial herb *Glaucium flavum* Crantz) were most abundant, and one (i.e. *Scrophularia canina* L. subsp. *bicolor* [Sm.] Greuter) was exclusively found outside *Genista* canopy (Table 2). Moreover, in all these cases, the effect of sampling area on plant biomass was not statistically significant (S11 Table). In the cases of *Bromus tectorum* L. subsp. *tectorum* and *Hieracium piloselloides* Vill., biomass in the OUT area was significantly higher than under the canopy, but only considering dead *Genista* individuals (Table 2).

## Discussion

Here, we demonstrate that *Genista* in a relatively short time-span (i.e. a few decades) can build-up an island of fertility under its canopy, by accumulating considerable C and N stocks below-ground and by improving the soil hydrological properties. Such changes in soil quality, coupled with the mitigation of harsh microclimatic conditions, lead to dramatic enhancement of plant colonization by both herbaceous and woody species. Moreover, we demonstrate that the capability of *Genista* shrubs to facilitate the establishment and growth of heterospecifics, including weedy and invasive species, showed a clear ontogenetic shift mediated by the amelioration of above- and below-ground conditions, including soil quality, temperature and hydrological regimes, as well as light attenuation through the canopy. As such, the effects of *Genista* individuals on coexisting species were size- and age-dependent, being mostly null for small-sized juveniles, and markedly facilitative for large adults. Then, our findings provide a possible mechanistic link between *Genista* invasion, biogenic changes in abiotic factors and plant facilitation.

### *Genista* effects on soil quality

The soil in the study area is a sandy, gravel rich, N poor substrate with an extremely low water holding capacity (Table 1). These soil characteristics, coupled with the physical instability and the very high surface temperature, are all factors hindering plant colonization. *Genista* is the only shrub that thrives in such harsh environmental conditions, whereas, noteworthy, the native nitrogen-fixing shrubs (*Cytisus scoparius* subsp. *scoparius* and *Spartium junceum*), as well as the invasive tree *Robinia pseudoacacia*, sporadically occur locally. In this context, *Genista* colonization dramatically altered the natural pedogenesis process leading, in less than 40 years, to an increase of topsoil C, N, and P stocks, up to one order of magnitude higher compared with outside the plant canopy. Our findings are consistent with Sanjurjo et al. [64], reporting a rapid pedogenesis under *Genista* canopy also at Mt. Etna in Sicily, where the species is native. Only few studies reported evidence concerning the time scale required for the built-up of fertility islands by nitrogen-fixing shrubs. Among these, Facelli and Brock [27] demonstrated that almost 150 years are required by *Acacia papyrocarpa* to significantly increase soil nutrient content in the arid climate of South Australia. On the other hand, colonization by *Alnus* species can drive to a relatively rapid N accumulation in primary succession after deglaciation [65] and river floodplain [66], as well as following volcanic eruptions [67]. The rapid accumulation of C and N stocks under *Genista* canopy can be related with the high amount of litterfall (~ 1 kg m^-2^ year^-1^ under adult plants) coupled with the specific chemical properties of such litter. Undecomposed *Genista* litter has a relatively high N concentration (2.1%) but, at the same time, a high lignin content (15.4%). It is well known that N content has a dual, contrasting effect on litter decomposition, stimulating during the early stages (up to 30–40% of mass loss) and limiting it thereafter [68]. At the beginning of litter decay process, high N content promotes microbial growth that rapidly consume labile C compounds, resulting in a high mass loss rate. In contrast, high N concentration at the later decomposition stages inhibits mass loss by favouring the formation of recalcitrant chemical complexes with lignin, though the microbiological and biochemical mechanisms have not yet fully clarified [69]. In fact, Berg et al. [70] reported that the limit value of litter decomposition (i.e. the amount of mass remaining at which the decay rate approaches zero) is highest for litters rich in N and lignin. In our decomposition experiment, *Genista* litter lost ~ 15% of its initial mass after 30 days of decomposition but only ~ 45% after 1 year. The initially rapid mass loss followed by a remarkable slow decomposition suggests a high limit value for the decomposition of this plant litter that, coupled with the high litterfall, could explain the rapid soil accumulation of C and N.

Classic ecosystem successional theory predicts that total P pool decreases over succession in the time scale of millennia [71]. Here, we showed that, in the early successional stage, *Genista* colonization drives to a rapid and remarkable increase of available P in the topsoil, likely as a result of the large litter input and recycling under the shrub canopy. Positive effects of nitrogen-fixing shrubs on available soil P have been previously reported for early primary successional stages, as in the cases of sand dunes [23] and semi-arid savannah [27]. The considerable organic C pool observed under adult *Genista* individuals can also explain the large increase of the cation exchange capacity. On the same line, the higher enzymatic activity (FDA) and respiration recorded under the canopy of living and dead adults, compared to corresponding open areas, likely reflect a high microbial activity in response to a larger amount of available organic substrates.

Generally, soil pH progressively declined during primary succession, likely as a consequence of accumulation of organic acids. Similar trends are more pronounced for glacial moraines, sand dunes, and floodplains compared to volcanoes, where the eruptive substrates often are already acidic [72]. Barren substrates at the Grand Cone slopes are already sub-acid, but after *Genista* colonization soil pH is further reduced by ~ 1.2 points. Interestingly, soils collected under adult *Genista* plants, both living and dead, once dried were strongly water repellent compared to soils from the outside areas. Values of the water drop penetration time > 1 h indicate that below-canopy soils are extremely hydrophobic [73]. It is well known that soil microbes, such as fairy-ring forming fungi [52,74], as well as the biochemical quality of soil organic matter [75] may contribute to produce soil water repellency. Previous studies reported that sandy texture, low pH and high organic C content are all factors that promote the development of soil water repellency [73,76]. The large increase of soil water repellency observed under living and dead *Genista* plants, compared with outside areas, is consistent with previous findings, since *Genista* during primary succession, greatly enhanced soil organic C accumulation and, at the same time, decreased soil pH of the sandy soil. The nature of the involved chemical compounds and the underlying molecular mechanisms are still unclear [77]. We acknowledge that the ecological relevance of such mechanism in open field remains to be proven, which would require further experimental studies.

Finally, topsoil under the canopy of older *Genista* individuals showed a significant increase of salinity (EC) compared to the outside areas. Such increase could be related to the release of mineral cations during organic matter mineralisation. However, the observed absolute EC values were well below those found in saline soils [78], suggesting that enhanced salinity is within the range of tolerance of most glycophytes.

### *Genista* effects on microclimate and soil hydrology

The study area is characterised by a Mediterranean climate with alternation of cold-wet and hot-dry seasons, with very short wet-to-dry and dry-to-wet transition stages. Several studies evidenced that Mediterranean summer drought establishes microclimatic conditions particularly severe for the regeneration of the herbaceous and shrub species, thus favouring high seedling and sapling mortality, also in mountain regions (e.g. [26,79,80]). These adverse microclimatic conditions are exacerbated on the bare soils of the study site, since the coarse soil texture allows for very limited water holding capacity, while the dark coloured and rough surface enhances the amount of soil incident radiation absorbed by the ground and transformed into heat [81]. The microclimatic conditions favourable for the vegetative growth in open areas are restricted to a very short wet-to-dry transition stage prior to the summer drought. The *Genista* canopy, by ameliorating the microclimatic conditions, likely extends the potential period favourable for the vegetative growth. The decrease of air temperature and the simultaneous increase of air relative humidity during the hottest hours of the summer days, may contribute to mitigate the vapour pressure deficit, the leaves temperature and the transpiration losses. Canopy shading also reduces the amount of incident solar radiation absorbed by the ground, mitigating soil heating and direct soil evaporation losses. All these mechanisms enhance seedling survival and growth by inducing a more favourable water balance in shrub and herbaceous seedlings (e.g. [26]).

The observed soil water content data also indicate an improvement of the soil water conditions under the *Genista* as compared with the open areas. Soil water content at 20 cm depth under the canopy exhibits larger values than outside during the entire year, probably as result of the soil organic carbon content of the soil at this depth under the canopy. The capacity of soil to store and supply water and air for plant growth is an important indicator of soil physical fertility [82]. The organic matter exerts a structure-forming effect on the soil which in turn increases the soil water retention [83], particularly in coarse-textured soils (as in the case herein examined) and at suction heads around those generally identified with the field capacity conditions [61].

The temporal patterns of the soil water content respectively observed underneath and outside the *Genista* canopy are especially different in the dry-to-wet transition stage. The soil water content outside the *Genista* reached high values very soon, usually within few hours, after the heavy rainfall events observed in early September (126 mm in 15 days). On the contrary, the soil moisture underneath the canopy increased more gradually during September and October, at both monitored depths. This long delay in soil wetting underneath the canopy could only be partly ascribed to the rainfall interception by the canopy and the litter layers, since the heavy rain events in early September largely exceeded the expected interception loss of the loose *Genista* canopy. The limited soil re-wetting after such precipitation events, observed under adult plants but not in the outside areas (Fig 4), can be explained by the soil water repellency, which hinders the wetting process starting from the dry conditions established during the summer season. As mentioned above, although the origin of water repellency in natural soils is poorly understood, it is commonly acknowledged that soil water repellency is caused by organic compounds attached on the surfaces of the mineral soil particles. The organic molecules tend to change their orientation when they come into contact with water and consequently the surface of soil particles becomes more hydrophilic [84]. Thus soil water repellency is variable in time and lasts for a finite time, from a few seconds to several days [73,84]. Recent studies confirmed that water repellency can enhance the hysteresis of the soil hydraulic properties, since it significantly modifies the soil water retention curves during the wetting process starting from initially dry soils, while it does not have any effect on the drainage curves of the soil water retention functions [85].

### Plant facilitation by *Genista*

We observed dramatic effects on plant diversity and distribution of the invaded ecosystem, mediated by the canopy of *Genista*. However, the capability of this nitrogen-fixing plant to facilitate heterospecifics showed a clear ontogenetic shift, modulated by plant age and size. It is known that seedlings and juveniles can not be effective nurse plants due to limited size and lifespan, which prevent from improving above-ground microclimatic conditions and persistently modify soil properties [19,86]. Accordingly, the effects of *Genista* on coexisting species were null for seedlings, and limited to an enhancement of standing litter for juveniles, but progressively turned into a prevailing facilitative pattern for living and dead adults. Facilitation by nitrogen-fixing plants has been often ascribed to an increase of water content and nutrients in the soil underlying plant canopy [23,25,35,87]. In fact, the facilitative role of nurse plants rely on their ability to alter the surrounding environment, thus enhancing seedling recruitment, plant growth and reproduction of potential beneficiaries [18]. Among the possible environmental modifications induced by living and dead *Genista* adults, changes in soil quality have likely played the main role, with contributions ameliorated microclimate both above and below ground. Consistently with previous findings, many plant species performed better under than outside *Genista* canopy, as related to the presence of islands of fertility. However, the selective distribution of some coexisting species, preferentially associated to living *Genista* individuals (e.g. *Briza maxima*, *Cynosurus echinatus*, and *Rumex scutatus* subsp. *scutatus*), while some others were most abundant under dead plants (e.g. *Avena barbata*, *Centranthus ruber* subsp. *ruber*, and *Lactuca serriola*), suggests that not a single factor drives the facilitative effect. Amelioration of micro-climatic conditions under *Genista* crowns is unlikely the main responsible mechanism of facilitation, because species richness was similar under living and dead plants, even if microclimatic conditions were significantly different in the two areas. In particular, the areas under dead *Genista* plants are characterized by higher PAR at ground level, as well as all along the vertical profile, thus showing intermediate microclimatic conditions with respect to the areas under living adults and areas outside the canopy.

Instead, distribution of living biomass and standing litter under living and dead *Genista* plants were discordant, with living biomass prevailing under dead *Genista* plants, and standing litter, as well as total biomass (i.e. living biomass + standing litter) under living adults. On one hand, such different patterns could be speculatively attributed to differences of soil properties between the two IN areas, such as microbial respiration and water repellency. On the other hand, considering the very similar pattern of most soil variables under living and dead plants, biomass differences could be most related to microclimatic differences. In this way, PAR attenuation under living adult plants is not likely to limit the overall plant growth, but instead could provide milder conditions compared to the area under dead plants. However, differences in light availability are likely an important factor to determine the distribution of several coexisting species under either living or dead *Genista* plants. In this regard, canopy of living adults provided small changes of air temperature and relative humidity, even during the hottest days of the year. On the other hand, *Genista* canopy was very effective in buffering the extremely high temperatures recorded at soil surface (up to +81°C) and in the topsoil, which outside the canopy persisted throughout the spring and summer seasons (e.g. in summer +35°C was reached almost every day at a soil depth of 5 cm). As a consequence, plant rooted under living adults of *Genista* have experienced milder temperatures and a higher moisture availability, at least during late spring and summer seasons. All these results indicate that *Genista* positively affected soil quality and hydrology properties, as well as microclimate, providing under its canopy a more suitable environment for plant colonization, thus acting as a nurse plant and facilitating several coexisting heterospecifics. In this regards, the hypothesis of soil quality improvement as a putative facilitation mechanism under adult *Genista* plants is further supported by the results of our greenhouse experiment. All target species, with the exception of *Genista* seedlings, performed significantly better when grown on soil collected under living and dead adults, compared to that from the outside areas, likely as a result of the higher nutrient contents. Although this general pattern was largely observed for all *Genista* heterospecifics, the magnitude of the plant-soil feedback effect mediated by *Genista* was species-specific. Interestingly, inter-specific differences were related to species occurrence along the ecological succession. In detail, *Briza maxima*, the most abundant annual grass in the IN zone, showed a remarkable positive response to IN soils (+634% compared to that of open area), while mid-successional *Fraxinus ornus* subsp. *ornus* (+120%), late-successional *Quercus ilex* subsp. *ilex* (+80%), and the invasive, nitrogen-fixing tree *Robinia pseudoacacia* (+79%) showed progressively lower growth increases on IN compared to OUT soils. These results suggest that the soil quality amelioration induced by *Genista* could allow the establishment and growth of several tree species once seed limitation is relaxed. Moreover, in spite of the fact that *Genista* seed fall was much higher in the area below the canopy (~ 512 and 13 seeds m^-2^ year^-1^ under and outside the canopy of adults, respectively), no seedlings occurred under the crown of both living or death plants, thus suggesting the self-inhibition of *Genista* recruitment under conspecifics due to a negative plant soil feedback [88,89,90]. This is supported by the bioassay where *Genista* seedlings were the less responsive to IN soils, with a similar growth recorded in OUT and IN substrates. This is particularly surprising because of the presence of islands of fertility under the crown area (Table 1). For instance, available P in the topsoil under the crown area was 6.6 times higher than outside the canopy, and a legume like *Genista* would normally thrive in such conditions. We acknowledge that the bioassay is phenomenological, then further studies are required to identify the factors underlying the observed *Genista* negative feedback. However, irrespective of the explanatory mechanisms, the occurrence of negative feedback towards the dominant species certainly further speeds up species replacement during the ecological succession [91,92].

## Conclusions

It is well established that plants can modify the surrounding biotic and abiotic environment, thus changing their own performance and those of coexisting species. As a consequence, the net effect of an invasive plant on coexisting species depends on their ability to alter the surrounding environment, according to their size, physiological status and life-span. Here, we found that the exotic shrub *Genista*, during its ontogenetic development, led in less than 40 years to a rapid development of fertility islands under its canopy with a substantial accumulation of C and N in the topsoil and increasing the availability of P, K^+^, Ca^2+^ and Mg^2+^. Moreover, *Genista* canopy dramatically affects soil hydrology and temperature regime. Such amelioration of soil quality, coupled with the mitigation of below-canopy microclimatic conditions, has enhanced plant colonization of the barren Grand Cone slopes, by both herbaceous and woody species. At community level, the spread of *Genista* fosters plant colonization and increases species diversity of native species, although some exotic species (e.g. *R*. *pseudoacacia*) can take advantage from the ameliorated environmental conditions. This could eventually drive to the spread of other, more resources-demanding exotic species [28], promoting alternative successional trajectories that may dramatically affect biodiversity. These data are the first to witness the invasion of *Genista aetnensis* outside of its origin area. In fact in a recent global review [93] report only *G*. *linifolia* L. and *G*. *monspessulana* (L.) L.A.S. Johnson as invaders. On the other hand, our study presents an additional example of the regime shifts that N-fixing shrubs can drive, particularly in the Mediterranean-climate regions [94,95], by reporting a detailed description of the soil quality and hydrology changes caused by *Genista aetnensis*, and linking them to possible effects on successional dynamics and ecosystem invasibility by further species. Further studies are needed to identity specific management practices that can limit the spread and impacts of *Genista aetnensis* and further invaders.

## Supporting Information

S1 Fig High-resolution time series of microclimate variables. Temporal profiles (10-minute data resolution) of air relative humidity and temperature and soil temperature at 5 cm and 20 cm depths outside (OUT—red lines) and under (IN_S3_—black lines) the canopy of living *Genista aetnensis* adult individuals during four consecutive days in three different periods of the year. The three periods are: 28 February – 3 March, soon after accumulated surface snow melt; 1–5 May, at the end of the rainy period in spring; 7–11 August, when the highest temperatures of the year have been recorded.
(TIF)

S2 Fig Effects of *Genista aetnensis* canopy on microclimate variables. Differences in air relative humidity and temperature between outside (OUT) and under the canopy of living (IN_S3_) *Genista aetnensis* adult individuals (black line, mean daily difference; red lines, 10^th^ and 90^th^ percentiles of the corresponding daily distributions retrieved from the 10-minutes time series).
(TIF)

S3 Fig Effects of *Genista aetnensis* canopy on soil surface temperature. Example of IR image (infrared radiation) taken at the Vesuvius Grand Cone. Image was collected in a sunny, summer day (13 August 2012, air temperature was +28°C) showing soil surface temperature outside (OUT) and under the canopy of a living *Genista aetnensis* adult individual (IN). Black dashed line indicates the edge of soil shaded by the shrub canopy.
(TIF)

S4 Fig Relationships between canopy effects on air temperature and relative humidity. Scatterplot of differences in air relative humidity and air temperature between the areas outside (OUT) and under the canopy of living (IN_S3_) *Genista aetnensis* adult individuals, during the period from May to August 2012 (black, regression line, Pearson’s correlation *r* = -0.86).
(TIF)

S1 Table Statistics on soil variables: normality tests. Summary of Shapiro-Wilk’s normality tests (W statistic and associated P-value) for soil variables under (IN) and outside (OUT) the canopy of *Genista* individuals.
(DOC)

S2 Table Statistics on soil variables: two-way ANOVA. Summary of the two-way ANOVA testing for main and interactive effects of ontogenetic stage of *Genista aetnensis* individuals (S1, S2, S3, D) and sampling area (either IN, under the canopy of *Genista*, or OUT, > 3 m from the canopy edge of the closest individual) on soil variables at the sampling sites.
(DOC)

S3 Table Soil variables outside *Genista aetnensis* canopy. Properties of topsoil (0–20 cm) in the survey area outside the canopy (OUT, > 3 m from the canopy edge of the closest individual) of *Genista aetnensis* individuals at four ontogenetic stages (S1, S2, S3, D). For each parameter, data refer to mean ± s.e.m. of 10 replicates. No significant differences were found among ontogenetic stages within the OUT area (Post-hoc Duncan test from GLM in S2 Table; *p* > 0.05 for all soil variables). For a comparison with data from the area under the canopy (IN), see Table 1 in the main text.
(DOC)

S4 Table Statistics on seed dispersal and litterfall. Summary of the two-way ANOVA testing for main and interactive effects of ontogenetic stage of *Genista aetnensis* individuals (IN_S1_, IN_S2_, IN_S3_, IN_D_) and sampling area (either IN, under the canopy of *Genista*, or OUT, > 3 m from the canopy edge of the closest individual) on *Genista* seed dispersal, *Genista* litterfall, and litterfall of heterospecifics at the sampling sites.
(DOC)

S5 Table Statistics on *Genista aetnenis* litter mass loss. Summary of the generalized linear model (GLM) testing for main and interactive effects of sampling area (either IN, under the canopy of adult *Genista aetnensis* individuals, or OUT, > 3 m from the canopy edge of the closest adult individual) and decomposition time (treated as a continuous variable) on litter mass of *Genista* decomposing at the field sites.
(DOC)

S6 Table Properties of *Genista aetnensis* decomposing litter. For each variable, data refer to mean ± s.e.m. of 10 replicated litterbags at each harvesting date. Data of chemical properties (cellulose, lignin, N, C-to-N and lignin-to-N ratios) are pooled for sampling areas (IN and OUT), since such factor did not affect significantly litter decomposition. Results of one-way ANOVA testing for the effect of decomposition time are also reported. Different letters indicate statistically significant time-dependent differences within each parameter (post-hoc Duncan test; *P*<0.05).
(DOC)

S7 Table Statistics on plant biomass data from the greenhouse bioassay. Summary of the two-way ANOVA testing for main and interactive effects of two soil treatments (i.e. factors used for selection of soil sampling sites) on the biomass of six plant species pot-grown in the greenhouse bioassay. In particular, tested effects were sampling area, either under or outside the canopy of *Genista aetnensis* individuals, and ontogenetic stage of the *Genista* individuals, either living or dead adults. See main text for details on experimental conditions.
(DOC)

S8 Table Statistics on photosynthetic active radiation (PAR). Summary of the generalized linear model (GLM) analysis of PAR at the Vesuvius Grand Cone. Data refer to testing for main and interactive effects of onthogenetic stage (S1, S2, S3, D) and sampling area (either under or outside the canopy) related to the closest *Genista* individual, and height above the ground.
(DOC)

S9 Table List of taxa sampled in the vegetation surveys. Biomass data of the 40 most frequent taxa (including mosses, considering aggregated data) were considered for data analysis, whereas less abundant taxa (marked with asterisk) were excluded from the analysis.
(DOC)

S10 Table Statistics on vegetation variables. Summary of the generalized linear mixed model (GLMM) analysis of total living biomass, standing litter, and species richness, from the vegetation surveys at the Vesuvius Grand Cone. Data refer to testing for main and interactive effects of onthogenetic stage (S1, S2, S3, D) and sampling area (either under or outside the canopy) related to the closest *Genista aetnensis* individual, sampling year (either 2010 or 2011) and growth season period (either May or August). Main and interactive random effects of the *Genista* individual, being not significant in all tested cases, are not shown to improve readability.
(DOC)

S11 Table Statistics on vegetation living biomass: generalized linear models (GLMs). Summary of the GLM analysis of the biomass of the 40 most abundant taxa recorded at the Vesuvius Grand Cone. Tested effects as in S10 Table.
(DOC)

S12 Table Statistics on vegetation living biomass: multiple t-tests. Living biomass of the most abundant taxa at the Vesuvius Grand Cone either under (IN) or outside (OUT) the canopy of *Genista aetnensis*. For each taxa, mean ± s.e.m. and results of testing for significant differences between the two sampling areas are reported (t-test for independent samples). Statistical results were considered significant at p<0.00125 (Bonferroni’s correction).
(DOC)

## References

[1] Pimentel D, Lach L, Zuniga R, Morrison D. Environmental and economic costs of nonindigenous species in the United States. BioScience. 2000;50: 53–65.

[2] Mooney HA, Hobbs R, editors. Invasive species in a changing world. Washington: Island Press; 2000.

[3] Daehler CC. The taxonomic distribution of invasive angiosperm plants: Ecological insights and comparison to agricultural weeds. Biol Conserv. 1998;84: 167–180.

[4] Vitousek PM, Walker LR. Biological invasion by *Myrica faya* in Hawai’i: plant demography, nitrogen fixation, ecosystem effects. Ecol Monogr. 1989;59: 247–265.

[5] Lonsdale WM. Rates of spread of an invading species—*Mimosa pigra* in northern Australia. J Ecol. 1993;81: 513–521.

[6] Pickart AJ, Miller LM, Duebendorfer TE. Yellow Bush Lupine invasion in Northern California coastal dunes I. Ecological impacts and manual restoration techniques. Restor Ecol. 1998;6: 59–68.

[7] Haubensak KA, Parker IM. Soil changes accompanying invasion of the exotic shrub *Cytisus scoparius* in glacial outwash prairies of western Washington [USA]. Plant Ecol. 2004;175: 71–79.

[8] Marchante E, Kjøller A, Struwe S, Freitas H. Short- and long-term impacts of *Acacia longifolia* invasion on the belowground processes of a Mediterranean coastal dune ecosystem. Appl Soil Ecol. 2008;40: 210–217.

[9] Castro SA, Figueroa JA, Muñoz-Schick M, Jaksic FM. Minimum residence time, biogeographical origin, and life cycle as determinants of the geographical extent of naturalized plants in continental Chile. Diversity Distrib. 2005;11: 183–191.

[10] DiTomaso JM, Healy EA. Weeds of California and Other Western States 1. Berkeley: University of California Department of Agriculture and Natural Resources Publications; 2007.

[11] Geerts S, Botha PW, Visser V, Richardson DM, Wilson JRU. Montpellier broom (*Genista monspessulana*) and Spanish broom (*Spartium junceum*) in South Africa: An assessment of invasiveness and options for management. S Afr J Bot. 2013;87: 134–145.

[12] D’Antonio CM, Vitousek PM. Biological invasion by exotic grasses, the grass/fire cycle, and global change. Annu Rev Ecol Syst. 1992;23: 63–87.

[13] Birken AS, Cooper DJ. Processes of *Tamarix* invasion and floodplain development along the lower Green River, Utah. Ecol Appl. 2006;16: 1103–1120. 16827006 10.1890/1051-0761(2006)016[1103:potiaf]2.0.co;2

[14] Ehrenfeld JG. Effects of exotic plant invasions on soil nutrient cycling processes. Ecosystems. 2003;6: 503–523.

[15] Levine JM, Vilà M, D’Antonio CM, Dukes JS, Grigulis K, Lavorel S. Mechanisms underlying the impacts of exotic plant invasions. Proc R Soc Lond B. 2003;270: 775–781. 10.1098/rspb.2003.2327 PMC1691311 12737654

[16] Belsky AJ, Mwonga SM, Amundson RG, Duxbury JM, Ali AR. Comparative effects of isolated trees on their undercanopy environments in high- and low-rainfall savannas. J Appl Ecol. 1993;30: 143–155.

[17] Schlesinger WH, Reynolds JF, Cunningham GL, Huenneke LF, Jarrell WM, Virginia RA, et al Biological feedbacks in global desertification. Science. 1990;247: 1043–1048. 17800060 10.1126/science.247.4946.1043

[18] Callaway RM. Positive interactions and interdependence in plant communities. Dordrecht: Springer; 2007.

[19] Bonanomi G, Incerti G, Mazzoleni S. Assessing occurrence, specificity, and mechanisms of plant facilitation in terrestrial ecosystems. Plant Ecol. 2011; 212: 1777–1790.

[20] Carrillo-Garcia Á, León De La Luz J-L, Bashan Y, Bethlenfalvay GJ. Nurse plants, mycorrhizae, and plant establishment in a disturbed area of the Sonoran desert. Restor Ecol. 1999;7: 321–335. 10644055

[21] Munzbergova Z, Ward D. *Acacia* trees as keystone species in Negev desert ecosystems. J Veg Sci. 2002;13: 227–236.

[22] Godínez-Alvarez H, Valiente-Banuet A. Germination and early seedling growth of Tehuacan Valley cacti species: the role of soils and seed ingestion by dispersers on seedling growth. J Arid Environ. 1998;39: 21–31. 9453711

[23] Bonanomi G, Rietkerk M, Dekker S, Mazzoleni S. Islands of fertility induce co-occurring negative and positive plant-soil feedbacks promoting coexistence. Plant Ecol. 2008;197: 207–218. 10.1111/j.1574-6941.2008.00550.x 18721146

[24] El-Keblawy A, Al-Rawai A. Impacts of the invasive exotic *Prosopis juliflora* (Sw.) D.C. on the native flora and soils of the UAE. Plant Ecol. 2007;190: 23–35.

[25] Moro MJ, Pugnaire FI, Haase P, Puigdefábregas J. Mechanisms of interaction between a leguminous shrub and its understorey in a semi-arid environment. Ecography. 1997;20: 175–184. 9216058

[26] Gómez-Aparicio L, Zamora R, Gómez JM, Hódar JA, Castro J, Baraza E. Applying plant facilitation to forest restoration: a meta-analysis of the use of shrubs as nurse plants. Ecol Appl. 2004;14: 1128–1138.

[27] Facelli JM, Brock DJ. Patch dynamics in arid lands: localized effects of *Acacia papyrocarpa* on soils and vegetation of open woodlands of south Australia. Ecography. 2000;23: 479–491.

[28] Davis MA, Grime JP, Thompson K. Fluctuating resources in plant communities: a general theory of invasibility. J Ecol. 2000;88: 528–534.

[29] Franco AC, Nobel PS. Effect of nurse plants on the microhabitat and growth of cacti. J Ecol. 1989;77: 870–886.

[30] Callaway RM. Positive interactions in plant communities and the individualistic-continuum concept. Oecologia. 1997;112: 143–149. 28307563 10.1007/s004420050293

[31] Cornejo FH, Varela A, Wright SJ. Tropical forest litter decomposition under seasonal drought: nutrient release, fungi and bacteria. Oikos. 1994;70: 183–190.

[32] Aguilera LE, Gutiérrez JR, Meserve PL. Variation in soil micro-organisms and nutrients underneath and outside the canopy of *Adesmia bedwellii* (Papilionaceae) shrubs in arid coastal Chile following drought and above average rainfall. J Arid Environ. 1999;42: 61–70.

[33] Miriti MN. Ontogenetic shift from facilitation to competition in a desert shrub. J Ecol. 2006;94: 973–979.

[34] Soliveres S, DeSoto L, Maestre FT, Olano JM. Spatio-temporal heterogeneity in abiotic factors modulate multiple ontogenetic shifts between competition and facilitation. Perspect. Plant Ecol, Evol Syst. 2010;12: 227–234.

[35] Maron JL, Connors PG. A native nitrogen-fixing shrub facilitates weed invasion. Oecologia. 1996;105: 302–312. 28307102 10.1007/BF00328732

[36] Agostini R. Alcuni reperti interessanti della flora della Campania. Delpinoa n s. 1959;1: 42–68.

[37] Agostini R. Vegetazione pioniera del Monte Vesuvio: aspetti fitosociologici ed evolutivi. Arch Bot e Biogeogr It. 1975;51: 11–34.

[38] Mazzoleni S, Ricciardi M, Aprile GG. Aspetti pionieri della vegetazione del Vesuvio. Ann Bot (Roma). 1989;47(Suppl. 6, Studi sul Territorio): 97–110.

[39] Mazzoleni S, Ricciardi M. Primary succession on the cone of Vesuvius In: Miles J, Walton DHW, editors. Primary succession on land. Oxford: Blackwell Scientific Publications Ltd; 1993 pp. 101–112.

[40] Motti R, Stinca A, Ricciardi M. Flora e Vegetazione In: Carpino F, Sammicheli F, editors. Laboratorio per il monitoraggio della biodiversità e cartografia del Parco Nazionale del Vesuvio. Napoli: Ente Parco Nazionale del Vesuvio; 2009 pp. 17–64.

[41] Ricciardi M, Aprile GG, La Valva V, Caputo G. (1988) La Flora del Somma-Vesuvio. Boll Soc Nat Napoli. 1988;96(1986): 3–121.

[42] Stinca A, Motti R. The vascular flora of the Royal Park of Portici (Naples, Italy). Webbia. 2009;64: 235–266.

[43] Stinca A, Motti R. Aggiornamenti floristici per il Somma-Vesuvio e l’Isola di Capri (Campania, Sud Italia). Inform Bot Ital. 2013;45: 35–43.

[44] Stinca A, D’Auria G, Motti R. *Manihot esculenta* (Euphorbiaceae), a new alien species in Italy. Hacquetia. 2014;13: 355–357. 10.1186/1475-2875-13-355 25199951 PMC4164714

[45] Pignatti S. Flora d’Italia 1–3. Bologna: Edagricole; 1982.

[46] Stinca A. Distribuzione, tassonomia ed impatto ecologico di specie aliene. Ph.D. Thesis, University of Naples Federico II, Italy. 2013.

[47] Bloem J, Hopkins DW, Benedetti A. Microbiological methods for assessing soil quality. Wallingford: CABI Publishing; 2006.

[48] Sparks DL, Page AL, Helmke PA, Loeppert RH, editors. Methods of Soil Analysis. Part 3-Chemical Methods SSSA Book Series 5. Madison: Soil Science Society of America and American Society of Agronomy; 1996.

[49] Loeppert RH, Suarez DL. Carbonate and gypsum In: Sparks DL, Page AL, Helmke PA, Loeppert RH, editors. Methods of Soil Analysis. Part 3-Chemical Methods. SSSA Book Series 5. Madison: Soil Science Society of America and American Society of Agronomy; 1996 pp. 437–474.

[50] Workneh F, Van Bruggen AHC, Drinkwater LE, Sherman C. Variables associated with corky root and *Phytophthora* root rot of tomatoes in organic and conventional farms. Phytopathology. 1993;83: 581–589.

[51] Alef K. Soil respiration In: Alef K, Nannipieri P, editors. Methods in Applied Soil Microbiology and Biochemistry. London: Academic Press; 1995 pp. 214–219.

[52] York CA, Canaway PM. Water repellent soils as they occur on UK golf greens. J Hydrol. 2000;231–232: 126–133.

[53] Berg B, McClaugherty C. Plant litter Decomposition, humus formation, carbon sequestration. Second edition Berlin: Springer; 2008.

[54] Gessner MO. Proximate lignin and cellulose In: Graça MAS, Bärlocher F, Gessner MO, editors. Methods to study litter decomposition. A Practical Guide. Dordrecht: Springer; 2005 pp. 115–120.

[55] Tutin TG, Heywood VH, Burges NA, Moore DM, Valentine DH, Walters SM, et al, editors. Flora Europaea 1–5. Cambridge: Cambridge University Press; 1964–1980. 10.1016/j.jep.2013.02.022

[56] Tutin TG, Burges NA, Chater AO, Edmondson JR, Heywood VH, Moore DM, et al, editors. Flora Europaea 1. 2nd ed Cambridge: Cambridge University Press; 1993.

[57] Castroviejo S, editor. Flora iberica: plantas vasculares de la Península Ibérica e Islas Baleares 1–8, 10–15, 17–18, 21. Madrid: Real Jardín Botánico, CSIC; 1986–2012.

[58] Conti F, Abbate G, Alessandrini A, Blasi C, editors. An Annotated Checklist of the Italian Vascular Flora. Roma: Palombi Editori; 2005.

[59] Conti F, Alessandrini A, Bacchetta G, Banfi E, Barberis G, Bartolucci F, et al Integrazioni alla checklist della flora vascolare italiana. Natura Vicentina. 2007;10(2006): 5–74.

[60] Gotelli NJ, Ellison AM. A primer of ecological statistics. Sunderland: Sinauer Associates; 2004.

[61] Romano N, Palladino M, Chirico GB. Parameterization of a bucket model for soil-vegetation-atmosphere modeling under seasonal climatic regimes. Hydrol Earth Syst Sc. 2011;15: 3877–3893.

[62] Chirico GB, Grayson RB, Western AW. A downward approach to identifying the structure and parameters of a process-based model for a small experimental catchment. Hydrol Process. 2003;17: 2239–2258.

[63] Piccolo F, Chirico GB. Sampling errors in rainfall measurements by weather radar. Adv Geo. 2005;2: 151–155.

[64] Sanjurjo MJF, Corti G, Cerini G, Ugolini FC. Pedogenesis induced by *Genista aetnensis* (Biv.) DC. on basaltic pyroclastic deposits at different altitudes, Mt. Etna, Italy. Geoderma 2003;115: 223–243.

[65] Chapin FS, Walker LR, Fastie CL, Sharman LC. Mechanisms of primary succession following deglaciation at Glacier Bay, Alaska. Ecol Monogr. 1994;64: 149–175.

[66] Nossov DR, Hollingsworth TN, Ruess RW, Kielland K. Development of *Alnus tenuifolia* stands on an Alaskan floodplain: patterns of recruitment, disease and succession. J Ecol. 2011;99: 621–633.

[67] Kamijo T, Kitayama K, Sugawara A, Urushimichi S, Sasai K. Primary succession of the warm-temperate broad-leaved forest on a volcanic island, Miyake-jima, Japan. Folia Geobot. 2002;37: 71–91.

[68] Bonanomi G, Incerti G, Giannino F, Mingo A, Lanzotti V, Mazzoleni S. Litter quality assessed by solid state ^13^C NMR spectroscopy predicts decay rate better than C/N and Lignin/N ratios. Soil Biol Biochem. 2013;56: 40–48.

[69] Hatakka A. Biodegradation of lignin In: Hofrichter M, Steinbüchel A, editors. Biopolymers 1. Lignin, humic substances and coal. Weinheim: Wiley; 2001 pp. 129–180.

[70] Berg B, Johansson M-B, Ekbohm G, McClaugherty C, Rutigliano F, Virzo De Santo A. Maximum decomposition limits of forest litter types: a synthesis. Can J Bot. 1996;74: 659–672.

[71] Walker TW, Syers JK. The fate of phosphorus during pedogenesis. Geoderma. 1976;15: 1–19.

[72] Walker LR, del Moral R. Primary succession and ecosystem rehabilitation. Cambridge: Cambridge University Press; 2003.

[73] Doerr SH, Shakesby RA, Walsh RPD. Soil water repellency: its causes, characteristics and hydro-geomorphological significance. Earth-Sci Rev. 2000;51: 33–65.

[74] Bonanomi G, Mingo A, Incerti G, Mazzoleni S, Allegrezza M. Fairy rings caused by a killer fungus foster plant diversity in species-rich grassland. J Veg Sci. 2012;23: 236–248.

[75] McGhie DA, Posner AM. The effect of plant top material on the water repellence of fired sands and water repellent soils. Aust. J Agr Res. 1981;32: 609–620.

[76] Mataix-Solera J, Arcenegui V, Guerrero C, Mayoral AM, Morales J, González J, et al Water repellency under different plant species in a calcareous forest soil in a semiarid Mediterranean environment. Hydrol Process. 2007;21: 2300–2309.

[77] Rillig MC. A connection between fungal hydrophobins and soil water repellency? Pedobiologia 2005;49: 395–399.

[78] Bonanomi G, D’Ascoli R, Antignani V, Capodilupo M, Cozzolino L, Marzaioli R, et al Assessing soil quality under intensive cultivation and tree orchards in Southern Italy. Appl Soil Ecol. 2011;47: 184–194.

[79] Rey PJ, Alcántara JM. Recruitment dynamics of a fleshy-fruited plant (*Olea europaea*): connecting patterns of seed dispersal to seedling establishment. J Ecol. 2000;88: 622–633.

[80] Castro J, Zamora R, Hódar JA, Gómez JM, Gómez-Aparicio L. Benefits of using shrubs as nurse plants for reforestation in Mediterranean mountains: a 4-year study. Restor Ecol. 2004;12: 352–358.

[81] Baumgardner MF, Silva LF, Biehl LL, Stoner ER. Reflectance properties of soils. Adv Agron. 1985;38: 1–44.

[82] Cass A. Interpretation of some soil physical indicators for assessing soil physical fertility In: Perverill KI, Sparrow LA, Reuter DJ, editors. Soil analysis: an interpretation manual. Melbourne: CSIRO Publishing; 1999 pp. 95–102.

[83] Rawls WJ, Pachepsky YA, Ritchie JC, Sobecki TM, Bloodworth H. Effect of soil organic carbon on soil water retention. Geoderma. 2003;116: 61–76.

[84] DeBano LF. Water Repellent Soils: a state-of-the-art. Berkeley: US Department of Agriculture, Forest Service, General Technical Report PSW-46; 1981.

[85] Diamantopoulos E, Durner W, Reszkowska A, Bachmann J. Effect of soil water repellency on soil hydraulic properties estimated under dynamic conditions. J Hydrol. 2013;486: 175–186.

[86] Incerti G, Giordano D, Stinca A, Senatore M, Termolino P, Mazzoleni S, et al Fire occurrence and tussock size modulate facilitation by *Ampelodesmos mauritanicus* . Acta Oecol. 2013;49: 116–124.

[87] Barnes PW, Archer S. Tree-shrub interactions in a subtropical savanna parkland: competition or facilitation? J Veg Sci. 1999;10: 525–536.

[88] Bever JD, Westover KM, Antonovics J. Incorporating the soil community into plant population dynamics: the utility of the feedback approach. J Ecol. 1997;85: 561–573.

[89] Mazzoleni S, Bonanomi G, Giannino F, Rietkerk M, Dekker SC, Zucconi F. Is plant biodiversity driven by decomposition processes? An emerging new theory on plant diversity. Community Ecol. 2007;8: 103–109. 17328789

[90] Kulmatisky A, Beard KH, Stevens JR, Cobbold SM. Plant-soil feedbacks: a meta analytical review. Ecol Lett. 2008;11: 980–992. 10.1111/j.1461-0248.2008.01209.x 18522641

[91] Van der Putten WH, Van Dijk C, Peters BAM. Plant-specific soil-borne diseases contribute to succession in foredune vegetation. Nature 1993;362: 53–56.

[92] Bonanomi G, Giannino F, Mazzoleni S. Negative plant-soil feedback and species coexistence. Oikos. 2005;111: 311–321.

[93] Rejmánek M, Richardson DM. Trees and shrubs as invasive alien species—2013 update of the global database. Diversity Distrib. 2013;19: 1093–1094.

[94] Gaertner M, Den Breeyen A, Hui C, Richardson DM. Impacts of alien plant invasions on species richness in Mediterranean-type ecosystems: a meta-analysis. Prog Phys Geogr. 2009;33: 319–338.

[95] Gaertner M, Biggs R, Te Beest M, Hui C, Molofsky J, Richardson DM. Invasive plants as drivers of regime shifts: identifying high-priority invaders that alter feedback relationships. Diversity Distrib. 2014;20: 733–744.

