# Supplementary figures and images for: Regime Shift by an Exotic Nitrogen-Fixing Shrub Mediates Plant Facilitation in Primary Succession
Source: PLoS One. 2015 Apr 2;10(4):e0123128. doi: 10.1371/journal.pone.0123128 (PMC4383633; doi:10.1371/journal.pone.0123128)

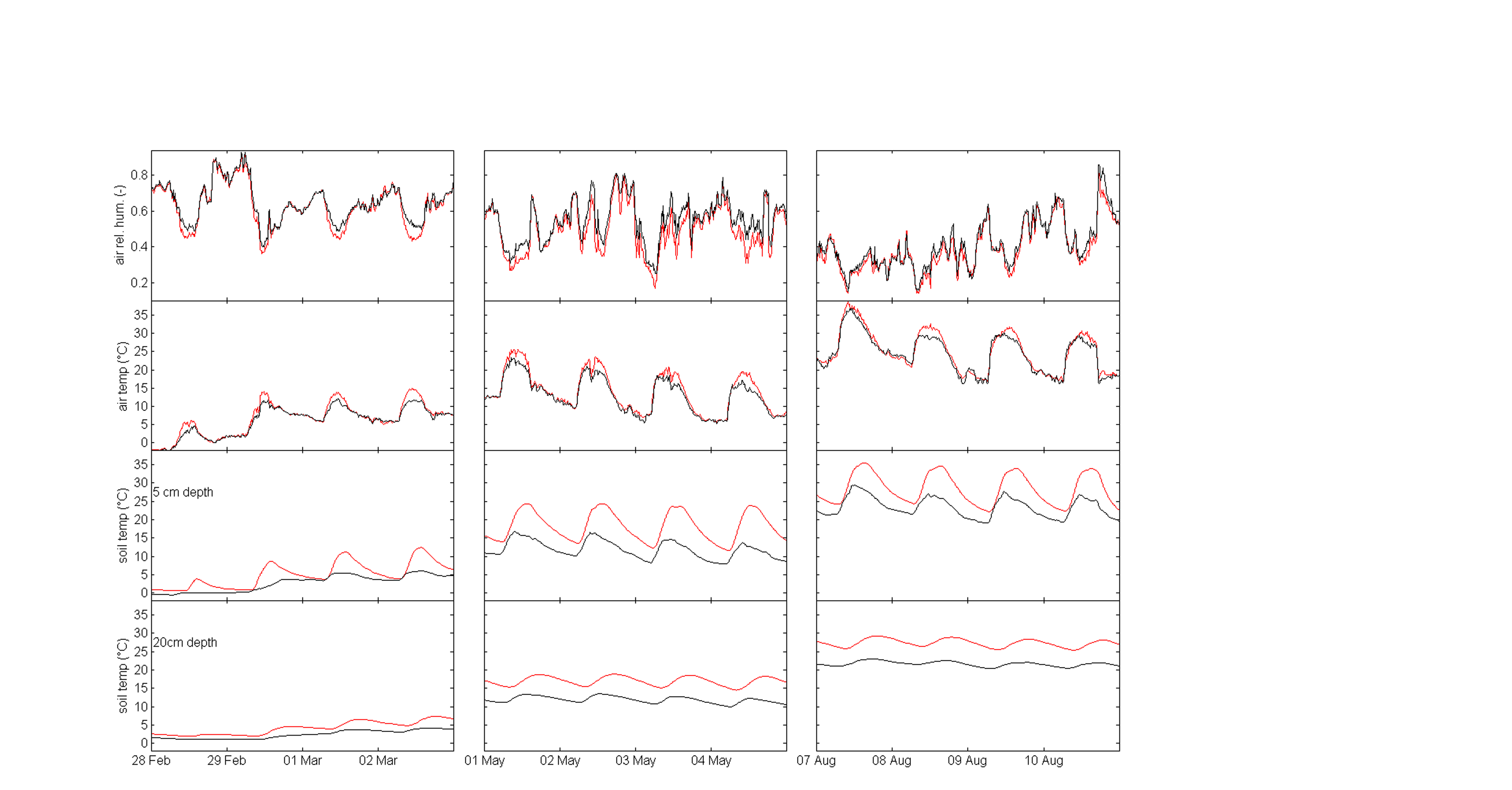

Supplement: S1 Fig — Temporal profiles (10-minute data resolution) of air relative humidity and temperature and soil temperature at 5 cm and 20 cm depths outside (OUT—red lines) and under (INS3—black lines) the canopy of living Genista aetnensis adult individuals during four consecutive days in three different periods of the year. The three periods are: 28 February – 3 March, soon after accumulated surface snow melt; 1–5 May, at the end of the rainy period in spring; 7–11 August, when the highest temperatures of the year have been recorded. (TIF) [file pone.0123128.s001.tif]

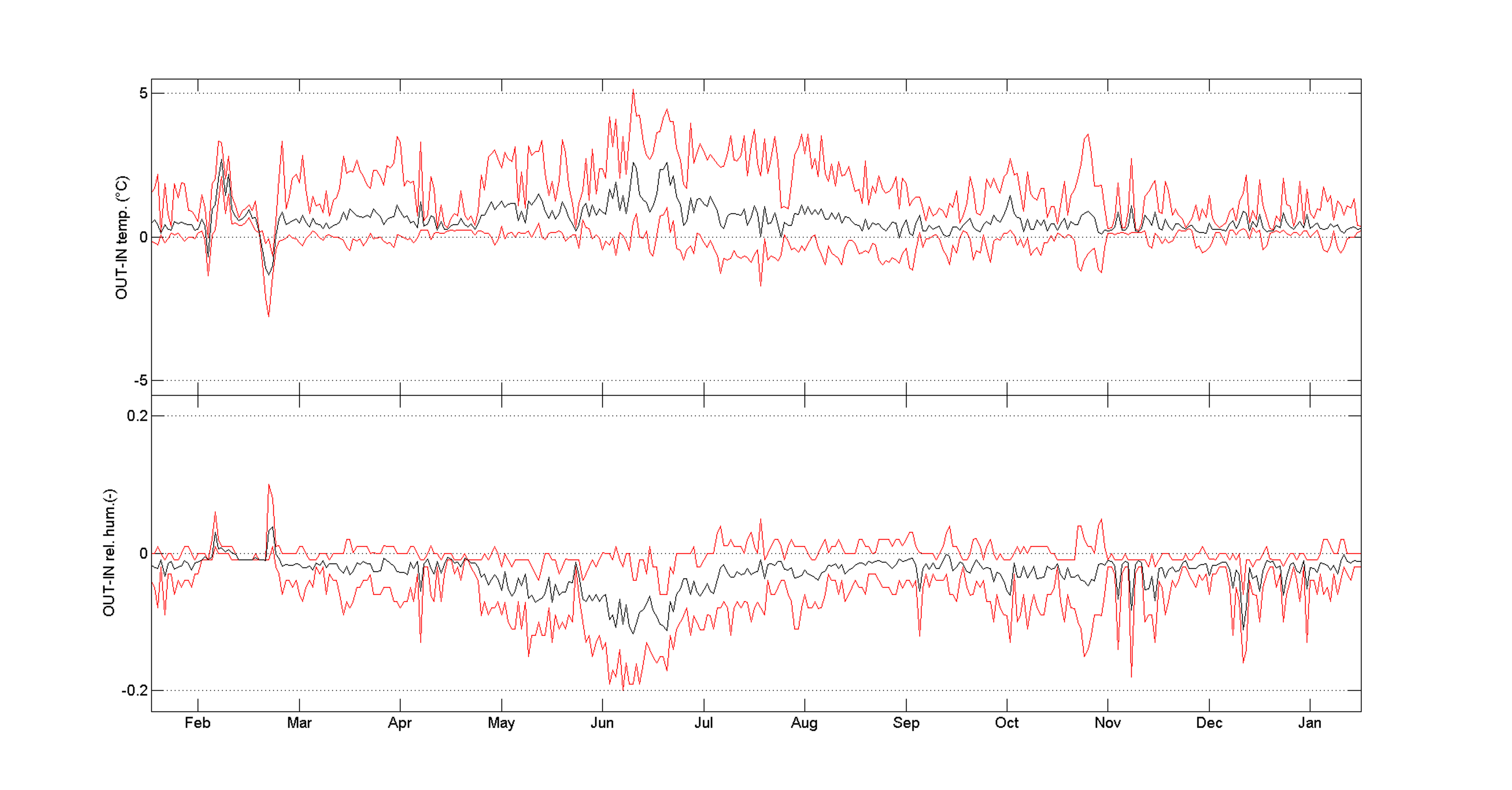

Supplement: S2 Fig — Differences in air relative humidity and temperature between outside (OUT) and under the canopy of living (INS3) Genista aetnensis adult individuals (black line, mean daily difference; red lines, 10th and 90th percentiles of the corresponding daily distributions retrieved from the 10-minutes time series). (TIF) [file pone.0123128.s002.tif]

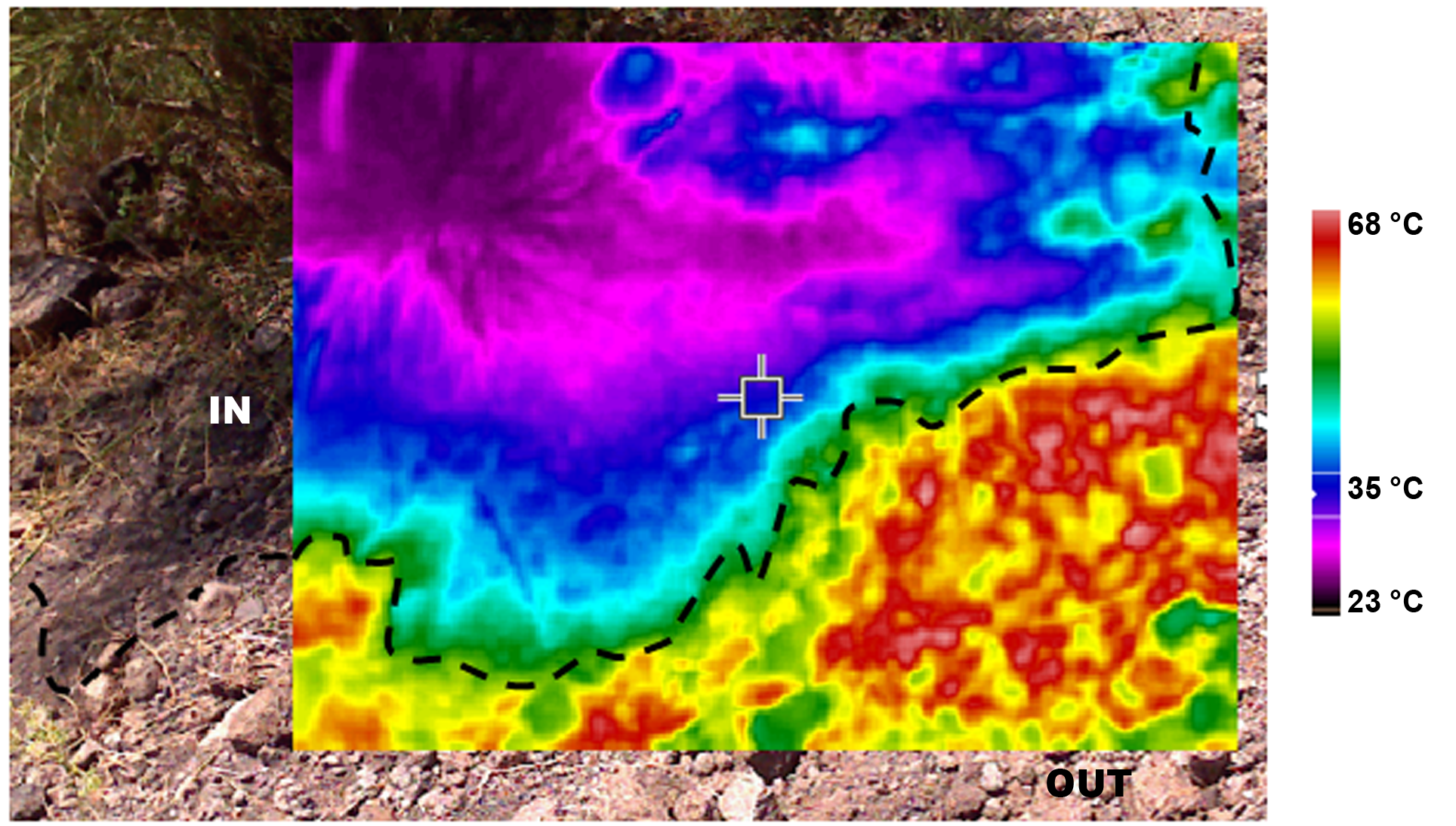

Supplement: S3 Fig — Example of IR image (infrared radiation) taken at the Vesuvius Grand Cone. Image was collected in a sunny, summer day (13 August 2012, air temperature was +28°C) showing soil surface temperature outside (OUT) and under the canopy of a living Genista aetnensis adult individual (IN). Black dashed line indicates the edge of soil shaded by the shrub canopy. (TIF) [file pone.0123128.s003.tif]

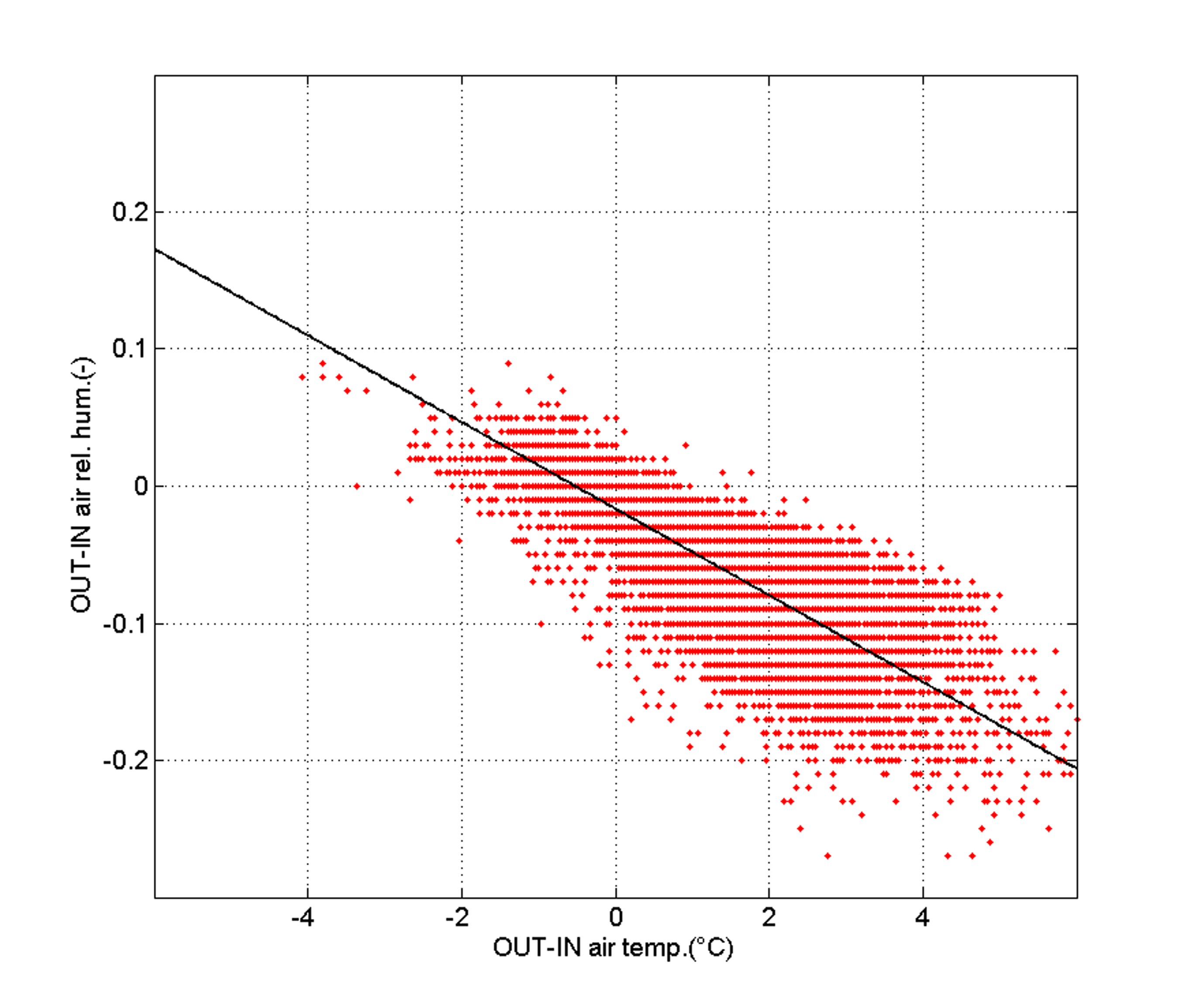

Supplement: S4 Fig — Scatterplot of differences in air relative humidity and air temperature between the areas outside (OUT) and under the canopy of living (INS3) Genista aetnensis adult individuals, during the period from May to August 2012 (black, regression line, Pearson’s correlation r = -0.86). (TIF) [file pone.0123128.s004.tif]
