# Supplementary material for: Regime Shift by an Exotic Nitrogen-Fixing Shrub Mediates Plant Facilitation in Primary Succession
Source: PLoS One. 2015 Apr 2;10(4):e0123128. doi: 10.1371/journal.pone.0123128 (PMC4383633; doi:10.1371/journal.pone.0123128)
Supplement: S1 Table — Summary of Shapiro-Wilk’s normality tests (W statistic and associated P-value) for soil variables under (IN) and outside (OUT) the canopy of Genista individuals. (DOC) [file pone.0123128.s005.doc]

**S1 Table. Statistics on soil variables: normality tests.** Summary of Shapiro-Wilk’s normality tests (W statistic and associated P-value) for soil variables under (IN) and outside (OUT) the canopy of *Genista* individuals.

| **Variable** | **IN** | | **OUT** | |
| --- | --- | --- | --- | --- |
|  | **W** | ***P*** | **W** | ***P*** |
| Texture |  |  |  |  |
| Fraction > 2 mm (mg g^-1^) | 0.9780 | 0.6837 | 0.9557 | 0.2165 |
| Fraction < 2 mm |  |  |  |  |
| Sand (mg g^-1^) | 0.9739 | 0.5569 | 0.9856 | 0.9138 |
| Silt (mg g^-1^) | 0.9698 | 0.4610 | 0.9730 | 0.5302 |
| Clay (mg g^-1^) | 0.8967 | ***< 0.005*** | 0.9061 | ***< 0.005*** |
| Organic C (mg g^-1^) | 0.8543 | ***< 0.005*** | 0.8631 | ***< 0.005*** |
| Total N (mg g^-1^) | 0.8585 | ***< 0.005*** | 0.7357 | ***< 0.005*** |
| C-to-N ratio | 0.9186 | ***< 0.005*** | 0.9327 | ***0.0317*** |
| P_2_O_5_ (mg kg^-1^) | 0.8706 | ***< 0.005*** | 0.7352 | ***< 0.005*** |
| pH | 0.9275 | ***0.0195*** | 0.8494 | ***< 0.005*** |
| Electrical conductivity (dS m^-1^) | 0.8611 | ***< 0.005*** | 0.8104 | ***< 0.005*** |
| CEC (meq+ 100 g^-1^) | 0.8403 | ***< 0.005*** | 0.8441 | ***< 0.005*** |
| K^+^ (meq+ 100 g^-1^) | 0.9382 | ***0.0454*** | 0.7627 | ***< 0.005*** |
| Mg^2+^ (meq+ 100 g^-1^) | 0.7964 | ***< 0.005*** | 0.7894 | ***< 0.005*** |
| Ca^2+^ (meq+ 100 g^-1^) | 0.8451 | ***< 0.005*** | 0.7930 | ***< 0.005*** |
| Na^+^ (meq+ 100 g^-1^) | 0.9495 | 0.1078 | 0.9641 | 0.3626 |
| Limestone | 0.9052 | ***< 0.005*** | 0.6529 | ***< 0.005*** |
| FDA (µg g^-1^ h^-1^) | 0.9754 | 0.6046 | 0.9529 | 0.1678 |
| Respiration (µg CO_2_-C g^-1^ soil h^-1^) | 0.9305 | ***0.0262*** | 0.8183 | ***< 0.005*** |
| Hydrophobicity (s) | 0.8514 | ***< 0.005*** | 0.8615 | ***< 0.005*** |
