# Supplementary material for: Regime Shift by an Exotic Nitrogen-Fixing Shrub Mediates Plant Facilitation in Primary Succession
Source: PLoS One. 2015 Apr 2;10(4):e0123128. doi: 10.1371/journal.pone.0123128 (PMC4383633; doi:10.1371/journal.pone.0123128)
Supplement: S2 Table — Summary of the two-way ANOVA testing for main and interactive effects of ontogenetic stage of Genista aetnensis individuals (S1, S2, S3, D) and sampling area (either IN, under the canopy of Genista, or OUT, > 3 m from the canopy edge of the closest individual) on soil variables at the sampling sites. (DOC) [file pone.0123128.s006.doc]

**S2 Table. Statistics on soil variables: two-way ANOVA.** Summary of the two-way ANOVA testing for main and interactive effects of ontogenetic stage of *Genista aetnensis* individuals (S1, S2, S3, D) and sampling area (either IN, under the canopy of *Genista*, or OUT, > 3 m from the canopy edge of the closest individual) on soil variables at the sampling sites.

|  | **SS** | **df** | **MS** | ***F*** | ***p*** |  |  | **SS** | **df** | **MS** | ***F*** | ***p*** |
| --- | --- | --- | --- | --- | --- | --- | --- | --- | --- | --- | --- | --- |
| **Fraction > 2mm** |  |  |  |  |  |  | **P_2_O_5_** |  |  |  |  |  |
| Stage (S) | 5973 | 3 | 1991 | 1.46 | 0.2326 |  | Stage (S) | 2966 | 3 | 989 | 10.46 | < 0.0001 |
| Sampling area (A) | 715 | 1 | 715 | 0.52 | 0.4716 |  | Sampling area (A) | 3607 | 1 | 3607 | 38.16 | < 0.0001 |
| S × A | 2340 | 3 | 780 | 0.57 | 0.6359 |  | S × A | 3125 | 3 | 1042 | 11.02 | < 0.0001 |
| **Sand** |  |  |  |  |  |  | **pH** |  |  |  |  |  |
| Stage (S) | 8118 | 3 | 2706 | 1.46 | 0.2321 |  | Stage (S) | 0.982 | 3 | 0.327 | 7.83 | 0.0001 |
| Sampling area (A) | 1162 | 1 | 1162 | 0.63 | 0.4310 |  | Sampling area (A) | 2.581 | 1 | 2.581 | 61.78 | < 0.0001 |
| S × A | 1881 | 3 | 627 | 0.34 | 0.7979 |  | S × A | 1.199 | 3 | 0.400 | 9.57 | < 0.0001 |
| **Silt** |  |  |  |  |  |  | **Electrical conductivity** |  |  |  |  |  |
| Stage (S) | 477.5 | 3 | 159.2 | 1.50 | 0.2212 |  | Stage (S) | 0.405 | 3 | 0.135 | 10.76 | < 0.0001 |
| Sampling area (A) | 360.4 | 1 | 360.4 | 3.39 | 0.0691 |  | Sampling area (A) | 0.431 | 1 | 0.431 | 34.41 | < 0.0001 |
| S × A | 143.5 | 3 | 47.8 | 0.45 | 0.7177 |  | S × A | 0.401 | 3 | 0.134 | 10.66 | < 0.0001 |
| **Clay** |  |  |  |  |  |  | **CEC** |  |  |  |  |  |
| Stage (S) | 1036 | 3 | 345 | 2.29 | 0.0850 |  | Stage (S) | 245.0 | 3 | 81.7 | 8.46 | 0.0001 |
| Sampling area (A) | 135 | 1 | 135 | 0.90 | 0.3466 |  | Sampling area (A) | 251.6 | 1 | 251.6 | 26.07 | < 0.0001 |
| S × A | 426 | 3 | 142 | 0.94 | 0.4254 |  | S × A | 244.8 | 3 | 81.6 | 8.46 | 0.0001 |
| **Organic C** |  |  |  |  |  |  | **K^+^** |  |  |  |  |  |
| Stage (S) | 2547 | 3 | 849 | 17.10 | < 0.0001 |  | Stage (S) | 0.106 | 3 | 0.035 | 8.31 | 0.0001 |
| Sampling area (A) | 2952 | 1 | 2952 | 59.44 | < 0.0001 |  | Sampling area (A) | 0.157 | 1 | 0.157 | 36.75 | < 0.0001 |
| S × A | 2614 | 3 | 871 | 17.55 | < 0.0001 |  | S × A | 0.125 | 3 | 0.042 | 9.76 | < 0.0001 |
| **Total N** |  |  |  |  |  |  | **Mg^2+^** |  |  |  |  |  |
| Stage (S) | 25.90 | 3 | 8.63 | 12.89 | < 0.0001 |  | Stage (S) | 2.282 | 3 | 0.761 | 4.92 | 0.0035 |
| Sampling area (A) | 27.99 | 1 | 27.99 | 41.80 | < 0.0001 |  | Sampling area (A) | 2.432 | 1 | 2.432 | 15.72 | 0.0002 |
| S × A | 26.53 | 3 | 8.84 | 13.20 | < 0.0001 |  | S × A | 2.200 | 3 | 0.733 | 4.74 | 0.0043 |
| **C-to-N ratio** |  |  |  |  |  |  | **Ca^2+^** |  |  |  |  |  |
| Stage (S) | 1.521 | 3 | 0.507 | 0.18 | 0.9090 |  | Stage (S) | 150.2 | 3 | 50.1 | 8.99 | < 0.0001 |
| Sampling area (A) | 0.001 | 1 | 0.001 | 0.01 | 0.9206 |  | Sampling area (A) | 154.9 | 1 | 154.9 | 27.83 | < 0.0001 |
| S × A | 4.931 | 3 | 1.644 | 0.59 | 0.6254 |  | S × A | 155.2 | 3 | 51.7 | 9.29 | < 0.0001 |
| **FDA** |  |  |  |  |  |  | **Na^+^** |  |  |  |  |  |
| Stage (S) | 34783 | 3 | 11594 | 0.50 | 0.6841 |  | Stage (S) | 0.0008 | 3 | 0.0003 | 0.96 | 0.4163 |
| Sampling area (A) | 108945 | 1 | 108945 | 4.69 | 0.0334 |  | Sampling area (A) | 0.0003 | 1 | 0.0003 | 1.25 | 0.2676 |
| S × A | 5407 | 3 | 1802 | 0.08 | 0.9720 |  | S × A | 0.0003 | 3 | 0.0001 | 0.38 | 0.7644 |
| **Respiration** |  |  |  |  |  |  | **Limestone** |  |  |  |  |  |
| Stage (S) | 6.202 | 3 | 2.067 | 9.84 | < 0.0001 |  | Stage (S) | 80.65 | 3 | 26.88 | 2.99 | 0.0362 |
| Sampling area (A) | 7.420 | 1 | 7.420 | 35.31 | < 0.0001 |  | Sampling area (A) | 41.08 | 1 | 41.08 | 4.56 | 0.0357 |
| S × A | 5.971 | 3 | 1.990 | 9.47 | < 0.0001 |  | S × A | 46.13 | 3 | 15.38 | 1.71 | 0.1722 |
| **Hydrophobicity** |  |  |  |  |  |  |  |  |  |  |  |  |
| Stage (S) | 143819 | 3 | 47939 | 58.71 | < 0.0001 |  |  |  |  |  |  |  |
| Sampling area (A) | 165982 | 1 | 165982 | 203.28 | < 0.0001 |  |  |  |  |  |  |  |
| S × A | 143819 | 3 | 47939 | 58.71 | < 0.0001 |  |  |  |  |  |  |  |
