# Supplementary material for: Regime Shift by an Exotic Nitrogen-Fixing Shrub Mediates Plant Facilitation in Primary Succession
Source: PLoS One. 2015 Apr 2;10(4):e0123128. doi: 10.1371/journal.pone.0123128 (PMC4383633; doi:10.1371/journal.pone.0123128)
Supplement: S3 Table — Properties of topsoil (0–20 cm) in the survey area outside the canopy (OUT, > 3 m from the canopy edge of the closest individual) of Genista aetnensis individuals at four ontogenetic stages (S1, S2, S3, D). For each parameter, data refer to mean ± s.e.m. of 10 replicates. No significant differences were found among ontogenetic stages within the OUT area (Post-hoc Duncan test from GLM in S2 Table; p > 0.05 for all soil variables). For a comparison with data from the area under the canopy (IN), see Table 1 in the main text. (DOC) [file pone.0123128.s007.doc]

**S3 Table. Soil variables outside *Genista aetnensis* canopy.** Properties of topsoil (0-20 cm) in the survey area outside the canopy (OUT, > 3 m from the canopy edge of the closest individual) of *Genista aetnensis* individuals at four ontogenetic stages (S1, S2, S3, D). For each parameter, data refer to mean ± s.e.m. of 10 replicates. No significant differences were found among ontogenetic stages within the OUT area (Post-hoc Duncan test from GLM in S2 Table; *p* > 0.05 for all soil variables). For a comparison with data from the area under the canopy (IN), see Table 1 in the main text.

| **Variable** | **OUT** | | | |
| --- | --- | --- | --- | --- |
|  | **S1** | **S2** | **S3** | **D** |
| Texture |  |  |  |  |
| Fraction > 2 mm (mg g^-1^) | 772±13 | 770±11 | 774±13 | 747±12 |
| Fraction < 2 mm |  |  |  |  |
| Sand (mg g^-1^) | 964±6 | 966±9 | 960±6 | 962±7 |
| Silt (mg g^-1^) | 15±5 | 17±4 | 23±8 | 11±4 |
| Clay (mg g^-1^) | 14±3 | 22±4 | 15±8 | 16±8 |
| Organic C (mg g^-1^) | 5.01±1.81 | 4.07±0.56 | 4.23±1.57 | 4.40±0.96 |
| Total N (mg g^-1^) | 0.52±0.10 | 0.41±0.12 | 0.61±0.22 | 0.32±0.18 |
| C-to-N ratio | 10.14±0.86 | 9.84±0.87 | 11.05±0.29 | 10.59±1.45 |
| P_2_O_5_ (mg kg^-1^) | 9.44±2.15 | 7.01±2.45 | 9.06±2.53 | 6.82±2.27 |
| pH | 6.39±0.08 | 6.54±0.10 | 6.45±0.14 | 6.51±0.13 |
| Electrical conductivity (dS m^-1^) | 0.053±0.006 | 0.045±0.005 | 0.052±0.007 | 0.048±0.007 |
| CEC (meq+ 100 g^-1^) | 2.19±0.26 | 2.16±0.25 | 2.36±0.12 | 2.06±0.18 |
| K^+^ (meq+ 100 g^-1^) | 0.21±0.02 | 0.22±0.03 | 0.27±0.11 | 0.16±0.09 |
| Mg^2+^ (meq+ 100 g^-1^) | 0.18±0.03 | 0.19±0.02 | 0.19±0.06 | 0.22±0.04 |
| Ca^2+^ (meq+ 100 g^-1^) | 1.53±0.27 | 1.51±0.27 | 1.62±0.21 | 1.31±0.10 |
| Na^+^ (meq+ 100 g^-1^) | 0.12±0.01 | 0.11±0.01 | 0.13±0.02 | 0.11±0.02 |
| Limestone | 1.67±1.03 | 2.32±1.66 | 2.45±1.25 | 3.12±1.86 |
| FDA (µg g^-1^ h^-1^) | 101±32 | 85±49 | 123±41 | 191±36 |
| Respiration (µg CO_2_-C g^-1^ soil h^-1^) | 1.39±0.14 | 1.55±0.24 | 1.07±0.14 | 1.26±0.24 |
| Hydrophobicity (s) | < 1±0 | < 1±0 | < 1±0 | < 1±0 |
