# Supplementary material for: Regime Shift by an Exotic Nitrogen-Fixing Shrub Mediates Plant Facilitation in Primary Succession
Source: PLoS One. 2015 Apr 2;10(4):e0123128. doi: 10.1371/journal.pone.0123128 (PMC4383633; doi:10.1371/journal.pone.0123128)
Supplement: S5 Table — Summary of the generalized linear model (GLM) testing for main and interactive effects of sampling area (either IN, under the canopy of adult Genista aetnensis individuals, or OUT, > 3 m from the canopy edge of the closest adult individual) and decomposition time (treated as a continuous variable) on litter mass of Genista decomposing at the field sites. (DOC) [file pone.0123128.s009.doc]

**S5 Table. Statistics on *Genista aetnenis* litter mass loss.** Summary of the generalized linear model (GLM) testing for main and interactive effects of sampling area (either IN, under the canopy of adult *Genista aetnensis* individuals, or OUT, > 3 m from the canopy edge of the closest adult individual) and decomposition time (treated as a continuous variable) on litter mass of *Genista* decomposing at the field sites.

|  | **SS** | **df** | **MS** | ***F*** | ***p*** |
| --- | --- | --- | --- | --- | --- |
| ***Genista* litter mass** |  |  |  |  |  |
| Sampling area (A) | 0.004 | 1 | 0.004 | 0.10 | 0.7526 |
| Decomposition time (T) | 52.154 | 1 | 52.154 | 1342.78 | < 0.0001 |
| A × T | 0.013 | 1 | 0.013 | 0.33 | 0.5651 |
| Error | 3.729 | 96 | 0.039 |  |  |
