# Supplementary material for: Regime Shift by an Exotic Nitrogen-Fixing Shrub Mediates Plant Facilitation in Primary Succession
Source: PLoS One. 2015 Apr 2;10(4):e0123128. doi: 10.1371/journal.pone.0123128 (PMC4383633; doi:10.1371/journal.pone.0123128)
Supplement: S6 Table — For each variable, data refer to mean ± s.e.m. of 10 replicated litterbags at each harvesting date. Data of chemical properties (cellulose, lignin, N, C-to-N and lignin-to-N ratios) are pooled for sampling areas (IN and OUT), since such factor did not affect significantly litter decomposition. Results of one-way ANOVA testing for the effect of decomposition time are also reported. Different letters indicate statistically significant time-dependent differences within each parameter (post-hoc Duncan test; P<0.05). (DOC) [file pone.0123128.s010.doc]

**S6 Table. Properties of *Genista* *aetnensis* decomposing litter.** For each variable, data refer to mean ± s.e.m. of 10 replicated litterbags at each harvesting date. Data of chemical properties (cellulose, lignin, N, C-to-N and lignin-to-N ratios) are pooled for sampling areas (IN and OUT), since such factor did not affect significantly litter decomposition. Results of one-way ANOVA testing for the effect of decomposition time are also reported. Different letters indicate statistically significant time-dependent differences within each parameter (post-hoc Duncan test; *P* < 0.05).

| ***Genista* litter** | **Decomposition time (days)** | | | | | **ANOVA** | |
| --- | --- | --- | --- | --- | --- | --- | --- |
|  | **0** | **30** | **90** | **180** | **360** | ***F*** | ***p*** |
| Litter mass (IN) | 5±0.001 e | 4.33±0.02 d | 4.07±0.02 c | 3.79±0.05 b | 2.70±0.05 a | 522.1 | < 0.0001 |
| Litter mass (OUT) | 5±0.001 e | 4.37±0.02 d | 4.07±0.02 c | 3.67±0.02 b | 2.81±0.03 a | 2172.6 | < 0.0001 |
| Cellulose (%) | 23.57±0.28 c | 23.63±0.21 c | 22.65±0.36 c | 16.17±0.24 b | 14.17±0.17 a | 107.4 | < 0.0001 |
| Lignin (%) | 15.45±0.21 a | 18.66±0.25 b | 22.26±0.22 c | 24.94±0.24 d | 29.27±0.23 e | 245.9 | < 0.0001 |
| N (%) | 2.11±0.01 a | 2.33±0.02 b | 2.48±0.07 b | 3.39±0.07 c | 4.17±0.08 d | 224.5 | < 0.0001 |
| C-to-N ratio | 23.79±0.06 e | 21.45±0.18 d | 18.83±0.45 c | 15.9±0.01 b | 12.32±0.21 a | 379.4 | < 0.0001 |
| Lignin-to-N ratio | 7.33±0.11 a | 8.02±0.22 b | 9.03±0.25 c | 7.39±0.19 a | 7.05±0.19 a | 16.2 | < 0.0001 |
