# Supplementary material for: Regime Shift by an Exotic Nitrogen-Fixing Shrub Mediates Plant Facilitation in Primary Succession
Source: PLoS One. 2015 Apr 2;10(4):e0123128. doi: 10.1371/journal.pone.0123128 (PMC4383633; doi:10.1371/journal.pone.0123128)
Supplement: S7 Table — Summary of the two-way ANOVA testing for main and interactive effects of two soil treatments (i.e. factors used for selection of soil sampling sites) on the biomass of six plant species pot-grown in the greenhouse bioassay. In particular, tested effects were sampling area, either under or outside the canopy of Genista aetnensis individuals, and ontogenetic stage of the Genista individuals, either living or dead adults. See main text for details on experimental conditions. (DOC) [file pone.0123128.s011.doc]

**S7 Table.** **Statistics on plant biomass data from the greenhouse bioassay.** Summary of the two-way ANOVA testing for main and interactive effects of two soil treatments (i.e. factors used for selection of soil sampling sites) on the biomass of six plant species pot-grown in the greenhouse bioassay. In particular, tested effects were sampling area, either under or outside the canopy of *Genista aetnensis* individuals, and ontogenetic stage of the *Genista* individuals, either living or dead adults. See main text for details on experimental conditions.

|  | **SS** | **df** | **MS** | ***F*** | ***p*** |
| --- | --- | --- | --- | --- | --- |
| ***Briza maxima*** |  |  |  |  |  |
| Stage (S) | 0.002 | 1 | 0.002 | 0.40 | 0.5296 |
| Sampling area (A) | 0.249 | 1 | 0.249 | 54.21 | < 0.0001 |
| S × A | 0.003 | 1 | 0.003 | 0.66 | 0.4184 |
| Error | 0.165 | 56 | 0.005 |  |  |
| ***Fraxinus ornus* subsp. *ornus*** |  |  |  |  |  |
| Stage (S) | 0.037 | 1 | 0.037 | 1.77 | 0.1891 |
| Sampling area (A) | 1.534 | 1 | 1.534 | 73.50 | < 0.0001 |
| S × A | 0.037 | 1 | 0.037 | 1.76 | 0.1904 |
| Error | 1.357 | 56 | 0.021 |  |  |
| ***Genista aetnensis*** |  |  |  |  |  |
| Stage (S) | 0.011 | 1 | 0.011 | 3.01 | 0.0880 |
| Sampling area (A) | 0.049 | 1 | 0.049 | 13.00 | 0.0007 |
| S × A | 0.001 | 1 | 0.001 | 0.21 | 0.6474 |
| Error | 0.315 | 56 | 0.004 |  |  |
| ***Quercus ilex* subsp. *ilex*** |  |  |  |  |  |
| Stage (S) | 1.321 | 1 | 1.321 | 3.07 | 0.0851 |
| Sampling area (A) | 7.912 | 1 | 7.912 | 18.40 | < 0.0001 |
| S × A | 0.043 | 1 | 0.043 | 0.10 | 0.7538 |
| Error | 29.671 | 56 | 0.430 |  |  |
| ***Robinia pseudoacacia*** | |  |  |  |  |
| Stage (S) | 0.002 | 1 | 0.002 | 0.18 | 0.6694 |
| Sampling area (A) | 0.409 | 1 | 0.409 | 40.43 | < 0.0001 |
| S × A | 0.000 | 1 | 0.000 | 0.01 | 0.9069 |
| Error | 0.667 | 56 | 0.010 |  |  |
| ***Spartium junceum*** |  |  |  |  |  |
| Stage (S) | 0.017 | 1 | 0.017 | 2.51 | 0.1185 |
| Sampling area (A) | 0.352 | 1 | 0.352 | 51.84 | < 0.0001 |
| S × A | 0.000 | 1 | 0.000 | 0.07 | 0.7872 |
| Error | 0.442 | 56 | 0.007 |  |  |
