# Supplementary material for: Regime Shift by an Exotic Nitrogen-Fixing Shrub Mediates Plant Facilitation in Primary Succession
Source: PLoS One. 2015 Apr 2;10(4):e0123128. doi: 10.1371/journal.pone.0123128 (PMC4383633; doi:10.1371/journal.pone.0123128)
Supplement: S8 Table — Summary of the generalized linear model (GLM) analysis of PAR at the Vesuvius Grand Cone. Data refer to testing for main and interactive effects of onthogenetic stage (S1, S2, S3, D) and sampling area (either under or outside the canopy) related to the closest Genista individual, and height above the ground. (DOC) [file pone.0123128.s012.doc]

**S8 Table. Statistics on photosynthetic active radiation (PAR).** Summary of the generalized linear model (GLM) analysis of PAR at the Vesuvius Grand Cone. Data refer to testing for main and interactive effects of onthogenetic stage (S1, S2, S3, D) and sampling area (either under or outside the canopy) related to the closest *Genista* individual, and height above the ground.

|  | **SS** | **df** | **MS** | ***F*** | ***p*** |
| --- | --- | --- | --- | --- | --- |
| Stage (S) | 10537.0 | 3 | 3512.3 | 35.1 | < 0.0001 |
| Sampling Area (A) | 57748.5 | 1 | 57748.5 | 577.5 | < 0.0001 |
| Height (H) | 9824.7 | 1 | 9824.7 | 98.2 | < 0.0001 |
| S × A | 23349.9 | 3 | 7783.3 | 77.8 | < 0.0001 |
| S × H | 1194.1 | 3 | 398.0 | 4.0 | 0.0092 |
| A × H | 9824.7 | 1 | 9824.7 | 98.2 | < 0.0001 |
| Error | 14700.6 | 147 | 100.0 |  |  |
