# Supplementary material for: Regime Shift by an Exotic Nitrogen-Fixing Shrub Mediates Plant Facilitation in Primary Succession
Source: PLoS One. 2015 Apr 2;10(4):e0123128. doi: 10.1371/journal.pone.0123128 (PMC4383633; doi:10.1371/journal.pone.0123128)
Supplement: S9 Table — Biomass data of the 40 most frequent taxa (including mosses, considering aggregated data) were considered for data analysis, whereas less abundant taxa (marked with asterisk) were excluded from the analysis. (DOC) [file pone.0123128.s013.doc]

**S9 Table. List of taxa sampled in the vegetation surveys.** Biomass data of the 40 most frequent taxa (including mosses, considering aggregated data) were considered for data analysis, whereas less abundant taxa (marked with asterisk) were excluded from the analysis.

| **Taxon** | |
| --- | --- |
| *Aira caryophyllea* L. subsp. *caryophyllea* | *Helichrysum litoreum* Guss.* |
| *Aira caryophyllea* L. subsp. *multiculmis* (Dumort.) Bonnier et Layens* | *Heliotropium europaeum* L.* |
| *Aira tenorii* Guss.* | *Hieracium piloselloides* Vill. |
| *Arabis collina* Ten. subsp. *collina* | *Hieracium racemosum* Willd. subsp. *crinitum* (Sm.) Rouy* |
| *Arabis collina* Ten. subsp. *rosea* (DC.) Minuto* | *Hypochaeris radicata* L. |
| *Arabis turrita* L. | *Lactuca muralis* (L.) Gaertn. |
| *Arrhenatherum elatius* (L.) P. Beauv. ex J. et C. Presl subsp. *elatius* | *Lactuca serriola* L. |
| *Artemisia campestris* L. subsp. *variabilis* (Ten.) Greuter | *Legousia falcata* (Ten.) Janch.* |
| *Asplenium onopteris* L.* | *Legousia speculum-veneris* (L.) Chaix* |
| *Avena barbata* L. | *Linaria purpurea* (L.) Mill. |
| *Avena lusitanica* (Tab. Morais) Baum* | *Lolium perenne* L.* |
| *Briza maxima* L. | *Myosotis arvensis* (L.) Hill subsp. *arvensis* |
| *Bromus sterilis* L. | *Myosotis ramosissima* Rochel ex Schult. subsp. *ramosissima** |
| *Bromus tectorum* L. subsp. *tectorum* | *Petrorhagia dubia* (Raf.) G. López et Romo |
| *Carduus pycnocephalus* L. subsp. *pycnocephalus* | *Picris hieracioides* L. subsp. *spinulosa* (Bertol. ex Guss.) Arcang. |
| *Centaurea deusta* Ten. | *Pinus nigra* J.F. Arnold subsp. *nigra* |
| *Centranthus ruber* (L.) DC. subsp. *ruber* | *Populus nigra* L.* |
| *Cerastium brachypetalum* Desp. ex Pers. subsp. *brachypetalum** | *Reichardia picroides* (L.) Roth* |
| *Cerastium glomeratum* Thuill.* | *Robinia pseudoacacia* L. |
| *Cerastium semidecandrum* L.* | *Rumex acetosella* L. subsp. *angiocarpus* (Murb.) Murb. |
| *Chondrilla juncea* L.* | *Rumex scutatus* L. subsp. *scutatus* |
| *Clematis vitalba* L. | *Scrophularia canina* L. subsp. *bicolor* (Sm.) Greuter |
| *Crepis leontodontoides* All.* | *Silene armeria* L.* |
| *Crepis neglecta* L.* | *Silene vulgaris* (Moench) Garcke subsp. *tenoreana* (Colla) Soldano et F. Conti |
| *Crepis setosa* Haller f.* | *Solidago virgaurea* L. subsp. *virgaurea* |
| *Cynosurus echinatus* L. | *Sonchus asper* (L.) Hill subsp. *asper* |
| *Cytisus scoparius* (L.) Link subsp. *scoparius* | *Stereocaulon vesuvianum* Pers. |
| *Dactylis glomerata* L. subsp. *glomerata* | *Torilis arvensis* (Huds.) Link subsp. *purpurea* (Ten.) Hayek* |
| *Daucus carota* L. subsp. *carota* | *Tragopogon porrifolius* L. subsp. *porrifolius** |
| *Draba muralis* L.* | *Trifolium arvense* L. subsp. *arvense* |
| *Elymus repens* (L.) Gould subsp. *repens** | *Urospermum dalechampii* (L.) F.W. Schmidt* |
| *Galium aparine* L. | *Vulpia ciliata* Dumort.* |
| *Geranium purpureum* Vill. | *Vulpia myuros* (L.) C.C. Gmel. |
| *Glaucium flavum* Crantz | Mosses (pooled data) |
