# Supplementary material for: Regime Shift by an Exotic Nitrogen-Fixing Shrub Mediates Plant Facilitation in Primary Succession
Source: PLoS One. 2015 Apr 2;10(4):e0123128. doi: 10.1371/journal.pone.0123128 (PMC4383633; doi:10.1371/journal.pone.0123128)
Supplement: S10 Table — Summary of the generalized linear mixed model (GLMM) analysis of total living biomass, standing litter, and species richness, from the vegetation surveys at the Vesuvius Grand Cone. Data refer to testing for main and interactive effects of onthogenetic stage (S1, S2, S3, D) and sampling area (either under or outside the canopy) related to the closest Genista aetnensis individual, sampling year (either 2010 or 2011) and growth season period (either May or August). Main and interactive random effects of the Genista individual, being not significant in all tested cases, are not shown to improve readability. (DOC) [file pone.0123128.s014.doc]

**S10 Table. Statistics on vegetation variables.** Summary of the generalized linear mixed model (GLMM) analysis of total living biomass, standing litter, and species richness, from the vegetation surveys at the Vesuvius Grand Cone. Data refer to testing for main and interactive effects of onthogenetic stage (S1, S2, S3, D) and sampling area (either under or outside the canopy) related to the closest *Genista aetnensis* individual, sampling year (either 2010 or 2011) and growth season period (either May or August). Main and interactive random effects of the *Genista* individual, being not significant in all tested cases, are not shown to improve readability.

|  | **SS** | **df** | **MS** | ***F*** | ***p*** |
| --- | --- | --- | --- | --- | --- |
| **Total living biomass** |  |  |  |  |  |
| Stage (S) | 5411449 | 3 | 1803816 | 109.32 | < 0.0001 |
| Sampling Area (A) | 2735280 | 1 | 2735280 | 165.77 | < 0.0001 |
| Year (Yr) | 358697 | 1 | 358697 | 21.74 | < 0.0001 |
| Season (Se) | 8678 | 1 | 8678 | 0.53 | 0.4686 |
| S × A | 2863620 | 3 | 954540 | 57.85 | < 0.0001 |
| S × Yr | 344184 | 3 | 114728 | 6.95 | 0.0001 |
| A × Yr | 42 | 1 | 42 | 0.00 | 0.9599 |
| S × Se | 5995 | 3 | 1998 | 0.12 | 0.9477 |
| A × Se | 12011 | 1 | 12011 | 0.73 | 0.3939 |
| Yr × Se | 12534 | 1 | 12534 | 0.76 | 0.3838 |
| **Standing litter** |  |  |  |  |  |
| Stage (S) | 21725241 | 3 | 7241747 | 115.91 | < 0.0001 |
| Sampling Area (A) | 34961314 | 1 | 34961314 | 559.59 | < 0.0001 |
| Year (Yr) | 84838 | 1 | 84838 | 1.36 | 0.2443 |
| Season (Se) | 32099 | 1 | 32099 | 0.51 | 0.4738 |
| S × A | 21573812 | 3 | 7191271 | 115.10 | < 0.0001 |
| S × Yr | 126629 | 3 | 42210 | 0.68 | 0.5672 |
| A × Yr | 51278 | 1 | 51278 | 0.82 | 0.3653 |
| S × Se | 117513 | 3 | 39171 | 0.63 | 0.5978 |
| A × Se | 17337 | 1 | 17337 | 0.28 | 0.5985 |
| Yr × Se | 12962 | 1 | 12962 | 0.21 | 0.6489 |
| **Species richness** |  |  |  |  |  |
| Stage (S) | 917.6 | 3 | 305.9 | 137.92 | < 0.0001 |
| Sampling Area (A) | 271.0 | 1 | 271.0 | 122.20 | < 0.0001 |
| Year (Yr) | 60.3 | 1 | 60.3 | 27.20 | < 0.0001 |
| Season (Se) | 27.8 | 1 | 27.8 | 12.55 | 0.0004 |
| S × A | 258.5 | 3 | 86.2 | 38.86 | < 0.0001 |
| S × Yr | 61.6 | 3 | 20.5 | 9.26 | < 0.0001 |
| A × Yr | 11.5 | 1 | 11.5 | 5.17 | 0.0233 |
| S × Se | 14.2 | 3 | 4.7 | 2.14 | 0.0943 |
| A × Se | 3.8 | 1 | 3.8 | 1.73 | 0.1890 |
| Yr × Se | 7.0 | 1 | 7.0 | 3.18 | 0.0751 |
