# Supplementary material for: Regime Shift by an Exotic Nitrogen-Fixing Shrub Mediates Plant Facilitation in Primary Succession
Source: PLoS One. 2015 Apr 2;10(4):e0123128. doi: 10.1371/journal.pone.0123128 (PMC4383633; doi:10.1371/journal.pone.0123128)
Supplement: S11 Table — Summary of the GLM analysis of the biomass of the 40 most abundant taxa recorded at the Vesuvius Grand Cone. Tested effects as in S10 Table. (DOC) [file pone.0123128.s015.doc]

**S11 Table. Statistics on vegetation living biomass: generalized linear models (GLMs).** Summary of the GLM analysis of the biomass of the 40 most abundant taxa recorded at the Vesuvius Grand Cone. Tested effects as in S10 Table.

|  | **SS** | **df** | **MS** | ***F*** | ***p*** |  |  | **SS** | **df** | **MS** | ***F*** | ***p*** |
| --- | --- | --- | --- | --- | --- | --- | --- | --- | --- | --- | --- | --- |
| ***Stereocaulon vesuvianum*** | |  |  |  |  |  | **Mosses (pooled data)** |  |  |  |  |  |
| Stage (S) | 42 | 3 | 14 | 0.64 | 0.5901 |  | Stage (S) | 184823 | 3 | 61608 | 21.56 | < 0.0001 |
| Sampling Area (A) | 144 | 1 | 144 | 6.59 | 0.0105 |  | Sampling Area (A) | 113633 | 1 | 113633 | 39.77 | < 0.0001 |
| Year (Yr) | 1 | 1 | 1 | 0.04 | 0.8427 |  | Year (Yr) | 5251 | 1 | 5251 | 1.84 | 0.1757 |
| Season (Se) | 17 | 1 | 17 | 0.78 | 0.3773 |  | Season (Se) | 3988 | 1 | 3988 | 1.40 | 0.2379 |
| S × A | 34 | 3 | 11 | 0.51 | 0.6738 |  | S × A | 173392 | 3 | 57797 | 20.23 | < 0.0001 |
| S × Yr | 72 | 3 | 24 | 1.10 | 0.3470 |  | S × Yr | 26468 | 3 | 8823 | 3.09 | 0.0267 |
| A × Yr | 0 | 1 | 0 | 0.00 | 0.9808 |  | A × Yr | 2571 | 1 | 2571 | 0.90 | 0.3432 |
| S × Se | 115 | 3 | 38 | 1.75 | 0.1561 |  | S × Se | 6810 | 3 | 2270 | 0.79 | 0.4972 |
| A × Se | 7 | 1 | 7 | 0.32 | 0.5745 |  | A × Se | 2125 | 1 | 2125 | 0.74 | 0.3888 |
| Yr × Se | 76 | 1 | 76 | 3.46 | 0.0632 |  | Yr × Se | 218 | 1 | 218 | 0.08 | 0.7827 |
| ***Aira caryophyllea* subsp. *caryophyllea*** |  |  |  |  |  |  | ***Arabis collina* subsp. *collina*** |  |  |  |  |  |
| Stage (S) | 17 | 3 | 6 | 1.79 | 0.1485 |  | Stage (S) | 11569 | 3 | 3856 | 3.04 | 0.0284 |
| Sampling Area (A) | 13 | 1 | 13 | 3.99 | 0.0461 |  | Sampling Area (A) | 6031 | 1 | 6031 | 4.76 | 0.0296 |
| Year (Yr) | 13 | 1 | 13 | 4.13 | 0.0425 |  | Year (Yr) | 680 | 1 | 680 | 0.54 | 0.4642 |
| Season (Se) | 13 | 1 | 13 | 4.18 | 0.0414 |  | Season (Se) | 112 | 1 | 112 | 0.09 | 0.7661 |
| S × A | 19 | 3 | 6 | 2.01 | 0.1114 |  | S × A | 14239 | 3 | 4746 | 3.74 | 0.0110 |
| S × Yr | 19 | 3 | 6 | 1.97 | 0.1175 |  | S × Yr | 3373 | 3 | 1124 | 0.89 | 0.4477 |
| A × Yr | 20 | 1 | 20 | 6.24 | 0.0127 |  | A × Yr | 909 | 1 | 909 | 0.72 | 0.3976 |
| S × Se | 19 | 3 | 6 | 1.97 | 0.1171 |  | S × Se | 733 | 3 | 244 | 0.19 | 0.9014 |
| A × Se | 20 | 1 | 20 | 6.20 | 0.0130 |  | A × Se | 6 | 1 | 6 | 0.00 | 0.9474 |
| Yr × Se | 18 | 1 | 18 | 5.58 | 0.0185 |  | Yr × Se | 1585 | 1 | 1585 | 1.25 | 0.2640 |
| ***Arabis turrita*** |  |  |  |  |  |  | ***Arrhenatherum elatius* subsp. *elatius*** | |  |  |  |  |
| Stage (S) | 8030 | 3 | 2677 | 3.11 | 0.0258 |  | Stage (S) | 255176 | 3 | 85059 | 8.47 | < 0.0001 |
| Sampling Area (A) | 2468 | 1 | 2468 | 2.87 | 0.0907 |  | Sampling Area (A) | 132201 | 1 | 132201 | 13.16 | 0.0003 |
| Year (Yr) | 61 | 1 | 61 | 0.07 | 0.7902 |  | Year (Yr) | 65129 | 1 | 65129 | 6.48 | 0.0111 |
| Season (Se) | 972 | 1 | 972 | 1.13 | 0.2880 |  | Season (Se) | 1904 | 1 | 1904 | 0.19 | 0.6635 |
| S × A | 6885 | 3 | 2295 | 2.67 | 0.0468 |  | S × A | 216509 | 3 | 72170 | 7.18 | 0.0001 |
| S × Yr | 167 | 3 | 56 | 0.06 | 0.9786 |  | S × Yr | 107490 | 3 | 35830 | 3.57 | 0.0140 |
| A × Yr | 44 | 1 | 44 | 0.05 | 0.8210 |  | A × Yr | 33934 | 1 | 33934 | 3.38 | 0.0666 |
| S × Se | 3119 | 3 | 1040 | 1.21 | 0.3055 |  | S × Se | 5192 | 3 | 1731 | 0.17 | 0.9151 |
| A × Se | 483 | 1 | 483 | 0.56 | 0.4539 |  | A × Se | 37 | 1 | 37 | 0.00 | 0.9514 |
| Yr × Se | 301 | 1 | 301 | 0.35 | 0.5541 |  | Yr × Se | 10776 | 1 | 10776 | 1.07 | 0.3008 |
| ***Artemisia campestris* subsp. *variabilis*** |  |  |  |  |  |  | ***Avena barbata*** |  |  |  |  |  |
| Stage (S) | 229 | 3 | 76 | 2.34 | 0.0724 |  | Stage (S) | 22054 | 3 | 7351 | 6.92 | 0.0001 |
| Sampling Area (A) | 151 | 1 | 151 | 4.62 | 0.0320 |  | Sampling Area (A) | 12615 | 1 | 12615 | 11.88 | 0.0006 |
| Year (Yr) | 175 | 1 | 175 | 5.36 | 0.0209 |  | Year (Yr) | 170 | 1 | 170 | 0.16 | 0.6896 |
| Season (Se) | 63 | 1 | 63 | 1.92 | 0.1667 |  | Season (Se) | 163 | 1 | 163 | 0.15 | 0.6950 |
| S × A | 292 | 3 | 97 | 2.98 | 0.0309 |  | S × A | 16012 | 3 | 5337 | 5.03 | 0.0019 |
| S × Yr | 197 | 3 | 66 | 2.01 | 0.1119 |  | S × Yr | 614 | 3 | 205 | 0.19 | 0.9013 |
| A × Yr | 148 | 1 | 148 | 4.54 | 0.0335 |  | A × Yr | 50 | 1 | 50 | 0.05 | 0.8277 |
| S × Se | 62 | 3 | 21 | 0.63 | 0.5962 |  | S × Se | 870 | 3 | 290 | 0.27 | 0.8448 |
| A × Se | 40 | 1 | 40 | 1.24 | 0.2667 |  | A × Se | 152 | 1 | 152 | 0.14 | 0.7058 |
| Yr × Se | 37 | 1 | 37 | 1.13 | 0.2871 |  | Yr × Se | 3336 | 1 | 3336 | 3.14 | 0.0768 |
| ***Briza maxima*** |  |  |  |  |  |  | ***Bromus sterilis*** |  |  |  |  |  |
| Stage (S) | 287914 | 3 | 95971 | 35.13 | < 0.0001 |  | Stage (S) | 8767 | 3 | 2922 | 3.65 | 0.0125 |
| Sampling Area (A) | 259329 | 1 | 259329 | 94.91 | < 0.0001 |  | Sampling Area (A) | 8541 | 1 | 8541 | 10.66 | 0.0012 |
| Year (Yr) | 11329 | 1 | 11329 | 4.15 | 0.0421 |  | Year (Yr) | 1083 | 1 | 1083 | 1.35 | 0.2454 |
| Season (Se) | 5 | 1 | 5 | 0.00 | 0.9656 |  | Season (Se) | 3684 | 1 | 3684 | 4.60 | 0.0324 |
| S × A | 239565 | 3 | 79855 | 29.23 | < 0.0001 |  | S × A | 8684 | 3 | 2895 | 3.61 | 0.0131 |
| S × Yr | 41486 | 3 | 13829 | 5.06 | 0.0018 |  | S × Yr | 4705 | 3 | 1568 | 1.96 | 0.1192 |
| A × Yr | 3027 | 1 | 3027 | 1.11 | 0.2929 |  | A × Yr | 489 | 1 | 489 | 0.61 | 0.4352 |
| S × Se | 16385 | 3 | 5462 | 2.00 | 0.1129 |  | S × Se | 3872 | 3 | 1291 | 1.61 | 0.1857 |
| A × Se | 152 | 1 | 152 | 0.06 | 0.8136 |  | A × Se | 4274 | 1 | 4274 | 5.33 | 0.0212 |
| Yr × Se | 758 | 1 | 758 | 0.28 | 0.5987 |  | Yr × Se | 1490 | 1 | 1490 | 1.86 | 0.1733 |
| ***Bromus tectorum* subsp. *tectorum*** |  |  |  |  |  |  | ***Carduus pycnocephalus* subsp. *pycnocephalus*** | |  |  |  |  |
| Stage (S) | 184 | 3 | 61 | 3.89 | 0.0090 |  | Stage (S) | 249 | 3 | 83 | 2.08 | 0.1016 |
| Sampling Area (A) | 98 | 1 | 98 | 6.22 | 0.0129 |  | Sampling Area (A) | 119 | 1 | 119 | 2.98 | 0.0848 |
| Year (Yr) | 244 | 1 | 244 | 15.54 | 0.0001 |  | Year (Yr) | 86 | 1 | 86 | 2.14 | 0.1438 |
| Season (Se) | 95 | 1 | 95 | 6.03 | 0.0143 |  | Season (Se) | 10 | 1 | 10 | 0.24 | 0.6253 |
| S × A | 130 | 3 | 43 | 2.76 | 0.0412 |  | S × A | 198 | 3 | 66 | 1.65 | 0.1764 |
| S × Yr | 185 | 3 | 62 | 3.92 | 0.0087 |  | S × Yr | 207 | 3 | 69 | 1.73 | 0.1598 |
| A × Yr | 112 | 1 | 112 | 7.15 | 0.0077 |  | A × Yr | 123 | 1 | 123 | 3.07 | 0.0802 |
| S × Se | 55 | 3 | 18 | 1.16 | 0.3247 |  | S × Se | 4 | 3 | 1 | 0.03 | 0.9925 |
| A × Se | 78 | 1 | 78 | 4.95 | 0.0264 |  | A × Se | 18 | 1 | 18 | 0.44 | 0.5066 |
| Yr × Se | 188 | 1 | 188 | 11.93 | 0.0006 |  | Yr × Se | 1 | 1 | 1 | 0.02 | 0.8834 |
| ***Centaurea deusta*** |  |  |  |  |  |  | ***Cetranthus ruber* subsp. *ruber*** |  |  |  |  |  |
| Stage (S) | 36.6 | 3 | 12.2 | 0.86 | 0.4593 |  | Stage (S) | 34193 | 3 | 11398 | 3.15 | 0.0245 |
| Sampling Area (A) | 0.7 | 1 | 0.7 | 0.05 | 0.8251 |  | Sampling Area (A) | 4601 | 1 | 4601 | 1.27 | 0.2598 |
| Year (Yr) | 25.3 | 1 | 25.3 | 1.79 | 0.1809 |  | Year (Yr) | 24240 | 1 | 24240 | 6.70 | 0.0099 |
| Season (Se) | 1.4 | 1 | 1.4 | 0.10 | 0.7555 |  | Season (Se) | 112 | 1 | 112 | 0.03 | 0.8605 |
| S × A | 67.5 | 3 | 22.5 | 1.59 | 0.1895 |  | S × A | 23347 | 3 | 7782 | 2.15 | 0.0926 |
| S × Yr | 31.8 | 3 | 10.6 | 0.75 | 0.5214 |  | S × Yr | 41844 | 3 | 13948 | 3.86 | 0.0094 |
| A × Yr | 1.1 | 1 | 1.1 | 0.08 | 0.7761 |  | A × Yr | 3435 | 1 | 3435 | 0.95 | 0.3302 |
| S × Se | 62.8 | 3 | 20.9 | 1.48 | 0.2182 |  | S × Se | 2343 | 3 | 781 | 0.22 | 0.8854 |
| A × Se | 32.1 | 1 | 32.1 | 2.27 | 0.1323 |  | A × Se | 63 | 1 | 63 | 0.02 | 0.8951 |
| Yr × Se | 2.5 | 1 | 2.5 | 0.18 | 0.6740 |  | Yr × Se | 749 | 1 | 749 | 0.21 | 0.6492 |
| ***Clematis vitalba*** |  |  |  |  |  |  | ***Cynosurus echinatus*** |  |  |  |  |  |
| Stage (S) | 5174.4 | 3 | 1724.8 | 2.581 | 0.0526 |  | Stage (S) | 3255 | 3 | 1085 | 3.07 | 0.0274 |
| Sampling Area (A) | 3470.2 | 1 | 3470.2 | 5.194 | 0.0230 |  | Sampling Area (A) | 1505 | 1 | 1505 | 4.25 | 0.0396 |
| Year (Yr) | 419.0 | 1 | 419.0 | 0.627 | 0.4287 |  | Year (Yr) | 125 | 1 | 125 | 0.35 | 0.5518 |
| Season (Se) | 0.5 | 1 | 0.5 | 0.001 | 0.9792 |  | Season (Se) | 213 | 1 | 213 | 0.60 | 0.4378 |
| S × A | 5174.4 | 3 | 1724.8 | 2.581 | 0.0526 |  | S × A | 3255 | 3 | 1085 | 3.07 | 0.0274 |
| S × Yr | 559.0 | 3 | 186.3 | 0.279 | 0.8407 |  | S × Yr | 130 | 3 | 43 | 0.12 | 0.9467 |
| A × Yr | 353.8 | 1 | 353.8 | 0.530 | 0.4671 |  | A × Yr | 126 | 1 | 126 | 0.36 | 0.5511 |
| S × Se | 27.2 | 3 | 9.1 | 0.014 | 0.9978 |  | S × Se | 2743 | 3 | 914 | 2.58 | 0.0523 |
| A × Se | 0.2 | 1 | 0.2 | 0.0002 | 0.9880 |  | A × Se | 664 | 1 | 664 | 1.88 | 0.1711 |
| Yr × Se | 2868.4 | 1 | 2868.4 | 4.293 | 0.0387 |  | Yr × Se | 134 | 1 | 134 | 0.38 | 0.5377 |
| ***Cytisus scoparius* subsp. *scoparius*** |  |  |  |  |  |  | ***Dactylis glomerata* subsp. *glomerata*** |  |  |  |  |  |
| Stage (S) | 2848 | 3 | 949 | 3.48 | 0.0158 |  | Stage (S) | 136 | 3 | 45 | 0.57 | 0.6371 |
| Sampling Area (A) | 149 | 1 | 149 | 0.55 | 0.4602 |  | Sampling Area (A) | 47 | 1 | 47 | 0.59 | 0.4439 |
| Year (Yr) | 1899 | 1 | 1899 | 6.96 | 0.0085 |  | Year (Yr) | 73 | 1 | 73 | 0.91 | 0.3412 |
| Season (Se) | 127 | 1 | 127 | 0.47 | 0.4946 |  | Season (Se) | 73 | 1 | 73 | 0.91 | 0.3412 |
| S × A | 158 | 3 | 53 | 0.19 | 0.9014 |  | S × A | 136 | 3 | 45 | 0.57 | 0.6371 |
| S × Yr | 2871 | 3 | 957 | 3.51 | 0.0152 |  | S × Yr | 219 | 3 | 73 | 0.91 | 0.4363 |
| A × Yr | 192 | 1 | 192 | 0.70 | 0.4024 |  | A × Yr | 40 | 1 | 40 | 0.50 | 0.4802 |
| S × Se | 1221 | 3 | 407 | 1.49 | 0.2159 |  | S × Se | 219 | 3 | 73 | 0.91 | 0.4363 |
| A × Se | 91 | 1 | 91 | 0.34 | 0.5629 |  | A × Se | 40 | 1 | 40 | 0.50 | 0.4802 |
| Yr × Se | 105 | 1 | 105 | 0.38 | 0.5360 |  | Yr × Se | 80 | 1 | 80 | 1.00 | 0.3186 |
| ***Daucus carota* subsp. *carota*** |  |  |  |  |  |  | ***Galium aparine*** |  |  |  |  |  |
| Stage (S) | 19167 | 3 | 6389 | 4.76 | 0.0027 |  | Stage (S) | 398 | 3 | 133 | 3.89 | 0.0089 |
| Sampling Area (A) | 8421 | 1 | 8421 | 6.28 | 0.0125 |  | Sampling Area (A) | 309 | 1 | 309 | 9.06 | 0.0027 |
| Year (Yr) | 9806 | 1 | 9806 | 7.31 | 0.0070 |  | Year (Yr) | 387 | 1 | 387 | 11.36 | 0.0008 |
| Season (Se) | 680 | 1 | 680 | 0.51 | 0.4768 |  | Season (Se) | 170 | 1 | 170 | 4.99 | 0.0259 |
| S × A | 8166 | 3 | 2722 | 2.03 | 0.1086 |  | S × A | 352 | 3 | 117 | 3.44 | 0.0166 |
| S × Yr | 15831 | 3 | 5277 | 3.93 | 0.0085 |  | S × Yr | 406 | 3 | 135 | 3.97 | 0.0081 |
| A × Yr | 3585 | 1 | 3585 | 2.67 | 0.1026 |  | A × Yr | 415 | 1 | 415 | 12.19 | 0.0005 |
| S × Se | 4227 | 3 | 1409 | 1.05 | 0.3696 |  | S × Se | 221 | 3 | 74 | 2.16 | 0.0918 |
| A × Se | 93 | 1 | 93 | 0.07 | 0.7923 |  | A × Se | 263 | 1 | 263 | 7.70 | 0.0057 |
| Yr × Se | 8 | 1 | 8 | 0.01 | 0.9382 |  | Yr × Se | 279 | 1 | 279 | 8.19 | 0.0043 |
| ***Geranium purpureum*** | |  |  |  |  |  | ***Glaucium flavum*** |  |  |  |  |  |
| Stage (S) | 1819 | 3 | 606 | 5.77 | 0.0007 |  | Stage (S) | 456 | 3 | 152 | 0.97 | 0.4076 |
| Sampling Area (A) | 940 | 1 | 940 | 8.95 | 0.0029 |  | Sampling Area (A) | 579 | 1 | 579 | 3.68 | 0.0555 |
| Year (Yr) | 503 | 1 | 503 | 4.79 | 0.0290 |  | Year (Yr) | 157 | 1 | 157 | 1.00 | 0.3186 |
| Season (Se) | 542 | 1 | 542 | 5.17 | 0.0234 |  | Season (Se) | 42 | 1 | 42 | 0.27 | 0.6049 |
| S × A | 971 | 3 | 324 | 3.08 | 0.0270 |  | S × A | 286 | 3 | 95 | 0.61 | 0.6112 |
| S × Yr | 509 | 3 | 170 | 1.61 | 0.1847 |  | S × Yr | 254 | 3 | 85 | 0.54 | 0.6553 |
| A × Yr | 328 | 1 | 328 | 3.13 | 0.0775 |  | A × Yr | 306 | 1 | 306 | 1.95 | 0.1632 |
| S × Se | 533 | 3 | 178 | 1.69 | 0.1672 |  | S × Se | 549 | 3 | 183 | 1.16 | 0.3227 |
| A × Se | 357 | 1 | 357 | 3.40 | 0.0657 |  | A × Se | 107 | 1 | 107 | 0.68 | 0.4101 |
| Yr × Se | 1038 | 1 | 1038 | 9.89 | 0.0017 |  | Yr × Se | 241 | 1 | 241 | 1.53 | 0.2165 |
| ***Hieracium piloselloides*** | |  |  |  |  |  | ***Hypochaeris radicata*** |  |  |  |  |  |
| Stage (S) | 8442 | 3 | 2814 | 8.75 | < 0.0001 |  | Stage (S) | 498 | 3 | 166 | 0.21 | 0.8912 |
| Sampling Area (A) | 721 | 1 | 721 | 2.24 | 0.1350 |  | Sampling Area (A) | 1155 | 1 | 1155 | 1.44 | 0.2298 |
| Year (Yr) | 519 | 1 | 519 | 1.61 | 0.2046 |  | Year (Yr) | 3462 | 1 | 3462 | 4.33 | 0.0378 |
| Season (Se) | 1170 | 1 | 1170 | 3.64 | 0.0569 |  | Season (Se) | 819 | 1 | 819 | 1.02 | 0.3117 |
| S × A | 4177 | 3 | 1392 | 4.33 | 0.0049 |  | S × A | 1884 | 3 | 628 | 0.79 | 0.5023 |
| S × Yr | 1681 | 3 | 560 | 1.74 | 0.1572 |  | S × Yr | 373 | 3 | 124 | 0.16 | 0.9262 |
| A × Yr | 1608 | 1 | 1608 | 5.00 | 0.0257 |  | A × Yr | 1203 | 1 | 1203 | 1.51 | 0.2203 |
| S × Se | 4176 | 3 | 1392 | 4.33 | 0.0049 |  | S × Se | 2324 | 3 | 775 | 0.97 | 0.4068 |
| A × Se | 904 | 1 | 904 | 2.81 | 0.0942 |  | A × Se | 349 | 1 | 349 | 0.44 | 0.5090 |
| Yr × Se | 597 | 1 | 597 | 1.86 | 0.1736 |  | Yr × Se | 1001 | 1 | 1001 | 1.25 | 0.2635 |
| ***Lactuca muralis*** |  |  |  |  |  |  | ***Lactuca serriola*** |  |  |  |  |  |
| Stage (S) | 30.1 | 3 | 10.0 | 0.71 | 0.5484 |  | Stage (S) | 12978 | 3 | 4326 | 1.85 | 0.1373 |
| Sampling Area (A) | 10.4 | 1 | 10.4 | 0.73 | 0.3927 |  | Sampling Area (A) | 5946 | 1 | 5946 | 2.54 | 0.1115 |
| Year (Yr) | 9.7 | 1 | 9.7 | 0.68 | 0.4091 |  | Year (Yr) | 5745 | 1 | 5745 | 2.45 | 0.1178 |
| Season (Se) | 9.7 | 1 | 9.7 | 0.68 | 0.4091 |  | Season (Se) | 319 | 1 | 319 | 0.14 | 0.7123 |
| S × A | 30.1 | 3 | 10.0 | 0.71 | 0.5484 |  | S × A | 12978 | 3 | 4326 | 1.85 | 0.1373 |
| S × Yr | 29.1 | 3 | 9.7 | 0.68 | 0.5623 |  | S × Yr | 12894 | 3 | 4298 | 1.84 | 0.1394 |
| A × Yr | 5.3 | 1 | 5.3 | 0.38 | 0.5403 |  | A × Yr | 3723 | 1 | 3723 | 1.59 | 0.2078 |
| S × Se | 29.1 | 3 | 9.7 | 0.68 | 0.5623 |  | S × Se | 2630 | 3 | 877 | 0.37 | 0.7715 |
| A × Se | 5.3 | 1 | 5.3 | 0.38 | 0.5403 |  | A × Se | 71 | 1 | 71 | 0.03 | 0.8617 |
| Yr × Se | 17.6 | 1 | 17.6 | 1.24 | 0.2656 |  | Yr × Se | 1193 | 1 | 1193 | 0.51 | 0.4756 |
| ***Linaria purpurea*** |  |  |  |  |  |  | ***Myosotis arvensis* subsp. *arvensis*** |  |  |  |  |  |
| Stage (S) | 51.5 | 3 | 17.2 | 0.40 | 0.7515 |  | Stage (S) | 138.6 | 3 | 46.2 | 3.96 | 0.0082 |
| Sampling Area (A) | 152.8 | 1 | 152.8 | 3.58 | 0.0590 |  | Sampling Area (A) | 22.7 | 1 | 22.7 | 1.95 | 0.1634 |
| Year (Yr) | 0.4 | 1 | 0.4 | 0.01 | 0.9274 |  | Year (Yr) | 30.7 | 1 | 30.7 | 2.63 | 0.1052 |
| Season (Se) | 6.0 | 1 | 6.0 | 0.14 | 0.7089 |  | Season (Se) | 36.7 | 1 | 36.7 | 3.15 | 0.0766 |
| S × A | 51.5 | 3 | 17.2 | 0.40 | 0.7515 |  | S × A | 97.3 | 3 | 32.4 | 2.78 | 0.0404 |
| S × Yr | 173.5 | 3 | 57.8 | 1.35 | 0.2559 |  | S × Yr | 56.0 | 3 | 18.7 | 1.60 | 0.1878 |
| A × Yr | 2.8 | 1 | 2.8 | 0.07 | 0.7978 |  | A × Yr | 1.6 | 1 | 1.6 | 0.13 | 0.7144 |
| S × Se | 159.5 | 3 | 53.2 | 1.25 | 0.2925 |  | S × Se | 70.2 | 3 | 23.4 | 2.01 | 0.1120 |
| A × Se | 24.7 | 1 | 24.7 | 0.58 | 0.4471 |  | A × Se | 2.9 | 1 | 2.9 | 0.25 | 0.6154 |
| Yr × Se | 56.9 | 1 | 56.9 | 1.33 | 0.2487 |  | Yr × Se | 86.9 | 1 | 86.9 | 7.45 | 0.0065 |
| ***Petrorhagia dubia*** |  |  |  |  |  |  | ***Picris hieracioides* subsp. *spinulosa*** |  |  |  |  |  |
| Stage (S) | 81.1 | 3 | 27.0 | 2.72 | 0.0435 |  | Stage (S) | 5816 | 3 | 1939 | 2.43 | 0.0642 |
| Sampling Area (A) | 0.3 | 1 | 0.3 | 0.03 | 0.8583 |  | Sampling Area (A) | 46 | 1 | 46 | 0.06 | 0.8106 |
| Year (Yr) | 4.9 | 1 | 4.9 | 0.49 | 0.4846 |  | Year (Yr) | 3514 | 1 | 3514 | 4.40 | 0.0362 |
| Season (Se) | 14.2 | 1 | 14.2 | 1.44 | 0.2314 |  | Season (Se) | 602 | 1 | 602 | 0.75 | 0.3853 |
| S × A | 10.6 | 3 | 3.5 | 0.36 | 0.7850 |  | S × A | 1729 | 3 | 576 | 0.72 | 0.5388 |
| S × Yr | 33.7 | 3 | 11.2 | 1.13 | 0.3357 |  | S × Yr | 5160 | 3 | 1720 | 2.16 | 0.0921 |
| A × Yr | 23.4 | 1 | 23.4 | 2.36 | 0.1252 |  | A × Yr | 960 | 1 | 960 | 1.20 | 0.2732 |
| S × Se | 2.3 | 3 | 0.8 | 0.08 | 0.9730 |  | S × Se | 1392 | 3 | 464 | 0.58 | 0.6272 |
| A × Se | 49.1 | 1 | 49.1 | 4.95 | 0.0264 |  | A × Se | 1389 | 1 | 1389 | 1.74 | 0.1875 |
| Yr × Se | 11.4 | 1 | 11.4 | 1.15 | 0.2840 |  | Yr × Se | 262 | 1 | 262 | 0.33 | 0.5669 |
| ***Pinus nigra* subsp. *nigra*** |  |  |  |  |  |  | ***Robinia pseudoacacia*** | |  |  |  |  |
| Stage (S) | 0.066 | 3 | 0.022 | 0.71 | 0.5482 |  | Stage (S) | 135.08 | 3 | 45.03 | 3.01 | 0.0298 |
| Sampling Area (A) | 0.018 | 1 | 0.018 | 0.59 | 0.4442 |  | Sampling Area (A) | 0.09 | 1 | 0.09 | 0.01 | 0.9392 |
| Year (Yr) | 0.022 | 1 | 0.022 | 0.72 | 0.3968 |  | Year (Yr) | 46.56 | 1 | 46.56 | 3.11 | 0.0784 |
| Season (Se) | 0.022 | 1 | 0.022 | 0.72 | 0.3968 |  | Season (Se) | 46.56 | 1 | 46.56 | 3.11 | 0.0784 |
| S × A | 0.066 | 3 | 0.022 | 0.71 | 0.5482 |  | S × A | 0.25 | 3 | 0.08 | 0.01 | 0.9994 |
| S × Yr | 0.062 | 3 | 0.021 | 0.67 | 0.5711 |  | S × Yr | 139.90 | 3 | 46.63 | 3.11 | 0.0258 |
| A × Yr | 0.032 | 1 | 0.032 | 1.02 | 0.3136 |  | A × Yr | 0.07 | 1 | 0.07 | 0.00 | 0.9439 |
| S × Se | 0.062 | 3 | 0.021 | 0.67 | 0.5711 |  | S × Se | 139.90 | 3 | 46.63 | 3.11 | 0.0258 |
| A × Se | 0.032 | 1 | 0.032 | 1.02 | 0.3136 |  | A × Se | 0.07 | 1 | 0.07 | 0.00 | 0.9439 |
| Yr × Se | 0.031 | 1 | 0.031 | 0.99 | 0.3190 |  | Yr × Se | 45.62 | 1 | 45.62 | 3.05 | 0.0814 |
| ***Rumex acetosella* subsp. *angiocarpus*** |  |  |  |  |  |  | ***Rumex scutatus* subsp. *scutatus*** |  |  |  |  |  |
| Stage (S) | 470.6 | 3 | 156.9 | 1.89 | 0.1296 |  | Stage (S) | 111632 | 3 | 37211 | 7.95 | < 0.0001 |
| Sampling Area (A) | 8.8 | 1 | 8.8 | 0.11 | 0.7451 |  | Sampling Area (A) | 21897 | 1 | 21897 | 4.68 | 0.0309 |
| Year (Yr) | 0.1 | 1 | 0.1 | 0.001 | 0.9807 |  | Year (Yr) | 15751 | 1 | 15751 | 3.37 | 0.0670 |
| Season (Se) | 165.8 | 1 | 165.8 | 2.00 | 0.1578 |  | Season (Se) | 127 | 1 | 127 | 0.03 | 0.8691 |
| S × A | 425.2 | 3 | 141.7 | 1.71 | 0.1637 |  | S × A | 54255 | 3 | 18085 | 3.86 | 0.0093 |
| S × Yr | 116.0 | 3 | 38.7 | 0.47 | 0.7057 |  | S × Yr | 7738 | 3 | 2579 | 0.55 | 0.6475 |
| A × Yr | 18.7 | 1 | 18.7 | 0.23 | 0.6353 |  | A × Yr | 108 | 1 | 108 | 0.02 | 0.8792 |
| S × Se | 374.0 | 3 | 124.7 | 1.50 | 0.2124 |  | S × Se | 17898 | 3 | 5966 | 1.27 | 0.2820 |
| A × Se | 1.4 | 1 | 1.4 | 0.02 | 0.8975 |  | A × Se | 1794 | 1 | 1794 | 0.38 | 0.5360 |
| Yr × Se | 113.4 | 1 | 113.4 | 1.37 | 0.2426 |  | Yr × Se | 2389 | 1 | 2389 | 0.51 | 0.4751 |
| ***Scrophularia canina* subsp. *bicolor*** |  |  |  |  |  |  | ***Silene vulgaris* subsp. *tenoreana*** |  |  |  |  |  |
| Stage (S) | 340 | 3 | 113 | 0.68 | 0.5657 |  | Stage (S) | 919 | 3 | 306 | 3.80 | 0.0102 |
| Sampling Area (A) | 712 | 1 | 712 | 4.27 | 0.0393 |  | Sampling Area (A) | 241 | 1 | 241 | 2.99 | 0.0843 |
| Year (Yr) | 226 | 1 | 226 | 1.35 | 0.2450 |  | Year (Yr) | 82 | 1 | 82 | 1.01 | 0.3145 |
| Season (Se) | 361 | 1 | 361 | 2.16 | 0.1418 |  | Season (Se) | 2 | 1 | 2 | 0.03 | 0.8670 |
| S × A | 340 | 3 | 113 | 0.68 | 0.5657 |  | S × A | 1296 | 3 | 432 | 5.35 | 0.0012 |
| S × Yr | 331 | 3 | 110 | 0.66 | 0.5770 |  | S × Yr | 50 | 3 | 17 | 0.21 | 0.8924 |
| A × Yr | 175 | 1 | 175 | 1.05 | 0.3058 |  | A × Yr | 4 | 1 | 4 | 0.05 | 0.8245 |
| S × Se | 298 | 3 | 99 | 0.60 | 0.6184 |  | S × Se | 247 | 3 | 82 | 1.02 | 0.3839 |
| A × Se | 337 | 1 | 337 | 2.02 | 0.1560 |  | A × Se | 156 | 1 | 156 | 1.93 | 0.1648 |
| Yr × Se | 91 | 1 | 91 | 0.54 | 0.4618 |  | Yr × Se | 179 | 1 | 179 | 2.22 | 0.1371 |
| ***Solidago virgaurea* subsp. *virgaurea*** |  |  |  |  |  |  | ***Sonchus asper* subsp. *asper*** |  |  |  |  |  |
| Stage (S) | 427 | 3 | 142 | 1.03 | 0.3803 |  | Stage (S) | 0.127 | 3 | 0.042 | 0.66 | 0.5793 |
| Sampling Area (A) | 138 | 1 | 138 | 1.00 | 0.3186 |  | Sampling Area (A) | 0.044 | 1 | 0.044 | 0.68 | 0.4101 |
| Year (Yr) | 121 | 1 | 121 | 0.87 | 0.3516 |  | Year (Yr) | 0.068 | 1 | 0.068 | 1.05 | 0.3057 |
| Season (Se) | 121 | 1 | 121 | 0.87 | 0.3516 |  | Season (Se) | 0.050 | 1 | 0.050 | 0.77 | 0.3799 |
| S × A | 427 | 3 | 142 | 1.03 | 0.3803 |  | S × A | 0.127 | 3 | 0.042 | 0.66 | 0.5793 |
| S × Yr | 452 | 3 | 151 | 1.09 | 0.3539 |  | S × Yr | 0.204 | 3 | 0.068 | 1.05 | 0.3688 |
| A × Yr | 142 | 1 | 142 | 1.02 | 0.3122 |  | A × Yr | 0.037 | 1 | 0.037 | 0.58 | 0.4474 |
| S × Se | 452 | 3 | 151 | 1.09 | 0.3539 |  | S × Se | 0.150 | 3 | 0.050 | 0.77 | 0.5092 |
| A × Se | 142 | 1 | 142 | 1.02 | 0.3122 |  | A × Se | 0.027 | 1 | 0.027 | 0.42 | 0.5149 |
| Yr × Se | 139 | 1 | 139 | 1.00 | 0.3175 |  | Yr × Se | 0.055 | 1 | 0.055 | 0.85 | 0.3576 |
| ***Trifolium arvense* subsp. *arvense*** |  |  |  |  |  |  | ***Vulpia myuros*** |  |  |  |  |  |
| Stage (S) | 927 | 3 | 309 | 3.57 | 0.0139 |  | Stage (S) | 2229 | 3 | 743 | 6.24 | 0.0004 |
| Sampling Area (A) | 761 | 1 | 761 | 8.79 | 0.0031 |  | Sampling Area (A) | 311 | 1 | 311 | 2.61 | 0.1064 |
| Year (Yr) | 786 | 1 | 786 | 9.08 | 0.0027 |  | Year (Yr) | 58 | 1 | 58 | 0.49 | 0.4854 |
| Season (Se) | 274 | 1 | 274 | 3.16 | 0.0759 |  | Season (Se) | 21 | 1 | 21 | 0.18 | 0.6718 |
| S × A | 220 | 3 | 73 | 0.85 | 0.4693 |  | S × A | 1591 | 3 | 530 | 4.45 | 0.0042 |
| S × Yr | 354 | 3 | 118 | 1.36 | 0.2534 |  | S × Yr | 67 | 3 | 22 | 0.19 | 0.9047 |
| A × Yr | 35 | 1 | 35 | 0.40 | 0.5259 |  | A × Yr | 428 | 1 | 428 | 3.60 | 0.0584 |
| S × Se | 110 | 3 | 37 | 0.42 | 0.7368 |  | S × Se | 208 | 3 | 69 | 0.58 | 0.6270 |
| A × Se | 54 | 1 | 54 | 0.62 | 0.4301 |  | A × Se | 238 | 1 | 238 | 2.00 | 0.1581 |
| Yr × Se | 147 | 1 | 147 | 1.70 | 0.1923 |  | Yr × Se | 101 | 1 | 101 | 0.85 | 0.3563 |
