# Supplementary material for: Regime Shift by an Exotic Nitrogen-Fixing Shrub Mediates Plant Facilitation in Primary Succession
Source: PLoS One. 2015 Apr 2;10(4):e0123128. doi: 10.1371/journal.pone.0123128 (PMC4383633; doi:10.1371/journal.pone.0123128)
Supplement: S12 Table — Living biomass of the most abundant taxa at the Vesuvius Grand Cone either under (IN) or outside (OUT) the canopy of Genista aetnensis. For each taxa, mean ± s.e.m. and results of testing for significant differences between the two sampling areas are reported (t-test for independent samples). Statistical results were considered significant at p<0.00125 (Bonferroni’s correction). (DOC) [file pone.0123128.s016.doc]

**S12 Table. Statistics on vegetation living biomass: multiple t-tests.** Living biomass of the most abundant taxa at the Vesuvius Grand Cone either under (IN) or outside (OUT) the canopy of *Genista aetnensis*. For each taxa, mean ± s.e.m. and results of testing for significant differences between the two sampling areas are reported (t-test for independent samples). Statistical results were considered significant at p < 0.00125 (Bonferroni’s correction).

| **Taxon** | **IN** | **OUT** | **t** | **df** | ***p*** |
| --- | --- | --- | --- | --- | --- |
| Mosses (pooled data) | 32.29±4.34 | 0.27±0.25 | 6.851 | 958 | **< 0.0001** |
| *Aira caryophyllea* subsp. *caryophyllea* | 0.01±0.01 | 0.34±0.15 | -2.306 | 958 | 0.0213 |
| *Arabis collina* subsp. *collina* | 9.39±2.55 | 1.70±0.67 | 2.738 | 958 | 0.0063 |
| *Arabis turrita* | 4.90±2.15 | 0.12±0.08 | 2.063 | 958 | 0.0394 |
| *Arrhenatherum elatius* subsp. *elatius* | 37.05±7.63 | 2.30±0.81 | 4.213 | 958 | **< 0.0001** |
| *Artemisia campestris* subsp. *variabilis* | 0.10±0.07 | 1.08±0.48 | -2.159 | 958 | 0.0311 |
| *Avena barbata* | 11.28±2.42 | 0.43±0.21 | 4.145 | 958 | **< 0.0001** |
| *Briza maxima* | 51.48±4.33 | 3.11±0.63 | 10.298 | 958 | **< 0.0001** |
| *Bromus sterilis* | 8.79±2.12 | 0.02±0.02 | 3.853 | 958 | **0.0001** |
| *Bromus tectorum* subsp. *tectorum* | 0.72±0.17 | 1.45±0.29 | -2.244 | 958 | 0.0251 |
| *Carduus pycnocephalus* subsp. *pycnocephalus* | 1.05±0.46 | 0.05±0.05 | 2.008 | 958 | 0.0449 |
| *Centaurea deusta* | 0.24±0.24 | 0.15±0.15 | 0.323 | 958 | 0.7470 |
| *Centranthus ruber* subsp. *ruber* | 10.46±4.38 | 2.99±0.95 | 1.556 | 958 | 0.1200 |
| *Clematis vitalba* | 5.58±1.89 | - | 2.740 | 958 | 0.0063 |
| *Cynosurus echinatus* | 3.68±1.38 | - | 2.471 | 958 | 0.0136 |
| *Cytisus scoparius* subsp. *scoparius* | 1.50±0.83 | 2.08±1.03 | -0.440 | 958 | 0.6599 |
| *Dactylis glomerata* subsp. *glomerata* | 0.65±0.65 | - | 0.929 | 958 | 0.3530 |
| *Daucus carota* subsp. *carota* | 10.12±2.67 | 1.09±0.70 | 3.065 | 958 | 0.0022 |
| *Galium aparine* | 1.72±0.45 | 0.07±0.05 | 3.415 | 958 | **0.0007** |
| *Geranium purpureum* | 3.38±0.77 | 0.36±0.11 | 3.611 | 958 | **0.0003** |
| *Glaucium flavum* | 0.15±0.15 | 2.01±1.04 | -1.895 | 958 | 0.0584 |
| *Hieracium piloselloides* | 2.08±0.66 | 3.12±1.37 | -0.714 | 958 | 0.4755 |
| *Hypochaeris radicata* | 0.92±0.60 | 3.69±2.27 | -1.255 | 958 | 0.2098 |
| *Lactuca muralis* | 0.31±0.27 | - | 1.038 | 958 | 0.2994 |
| *Lactuca serriola* | 7.31±3.55 | - | 1.915 | 958 | 0.0558 |
| *Linaria purpurea* | 1.06±0.47 | - | 2.077 | 958 | 0.0380 |
| *Myosotis arvensis* subsp. *arvensis* | 0.61±0.25 | 0.13±0.05 | 1.755 | 958 | 0.0796 |
| *Petrorhagia dubia* | 0.43±0.15 | 0.50±0.21 | -0.282 | 958 | 0.7782 |
| *Picris hieracioides* subsp. *spinulosa* | 4.86±1.64 | 3.07±1.46 | 0.805 | 958 | 0.4208 |
| *Pinus nigra* subsp. *nigra* | - | 0.01±0.01 | -1.076 | 958 | 0.2821 |
| *Robinia pseudoacacia* | 0.31±0.22 | 0.21±0.21 | 0.322 | 958 | 0.7474 |
| *Rumex acetosella* subsp. *angiocarpus* | 1.27±0.54 | 1.40±0.44 | -0.170 | 958 | 0.8653 |
| *Rumex scutatus* subsp. *scutatus* | 23.52±4.87 | 8.63±1.70 | 2.722 | 958 | 0.0066 |
| *Scrophularia canina* subsp. *bicolor* | - | 2.32±1.09 | -2.300 | 958 | 0.0217 |
| *Silene vulgaris* subsp. *tenoreana* | 1.85±0.61 | 0.31±0.31 | 2.164 | 958 | 0.0307 |
| *Solidago virgaurea* subsp. *virgaurea* | 0.86±0.86 | - | 0.929 | 958 | 0.3530 |
| *Sonchus asper* subsp. *asper* | 0.02±0.02 | - | 0.999 | 958 | 0.3178 |
| *Stereocaulon vesuvianum* | 0.11±0.08 | 1.06±0.38 | -2.619 | 958 | 0.0090 |
| *Trifolium arvense* subsp. *arvense* | 1.57±0.38 | 3.79±0.66 | -3.001 | 958 | 0.0028 |
| *Vulpia myuros* | 2.93±0.74 | 1.19±0.38 | 1.993 | 958 | 0.0466 |
